# Supplementary figures and images for: Identification and validation of calcium extrusion-related genes prognostic signature in colon adenocarcinoma
Source: PeerJ. 2024 Jul 10;12:e17582. doi: 10.7717/peerj.17582 (PMC11246022; doi:10.7717/peerj.17582)

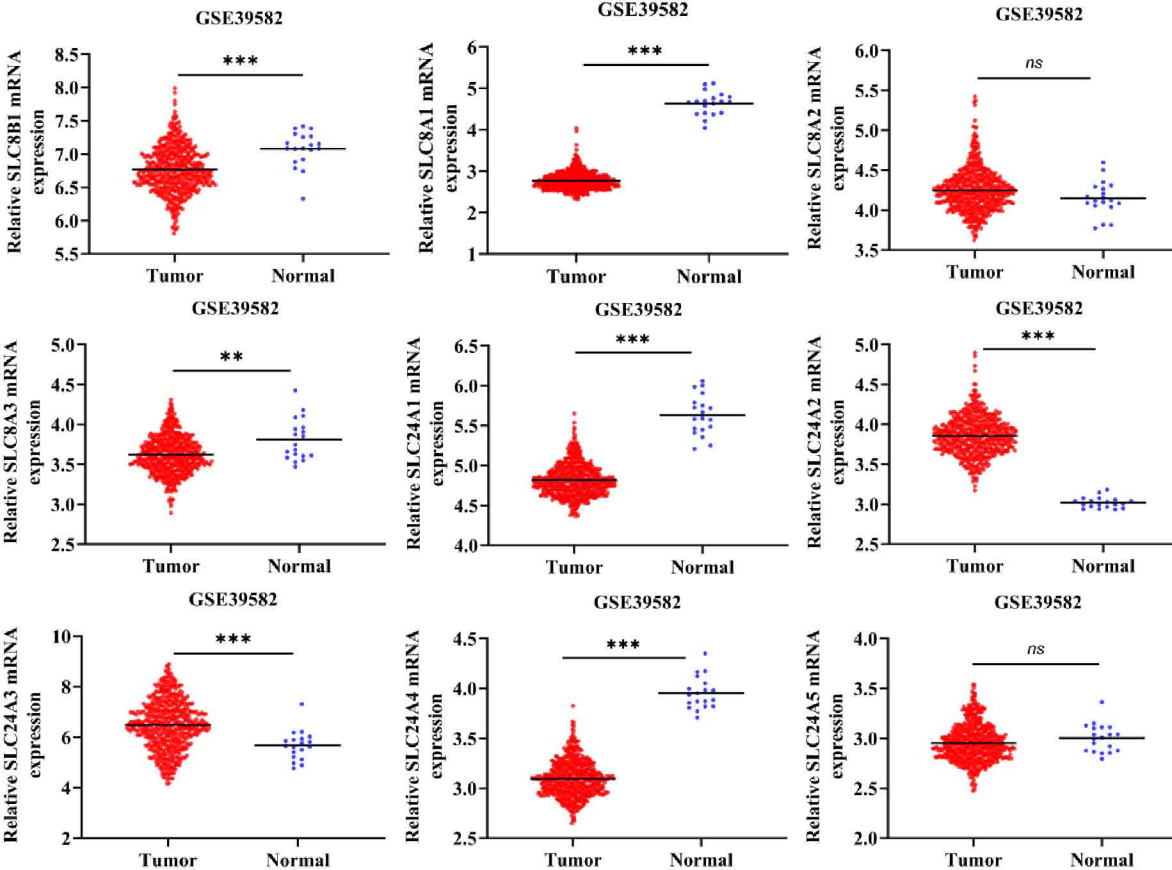

Supplement: Figure S1 [file peerj-12-17582-s001.pdf]

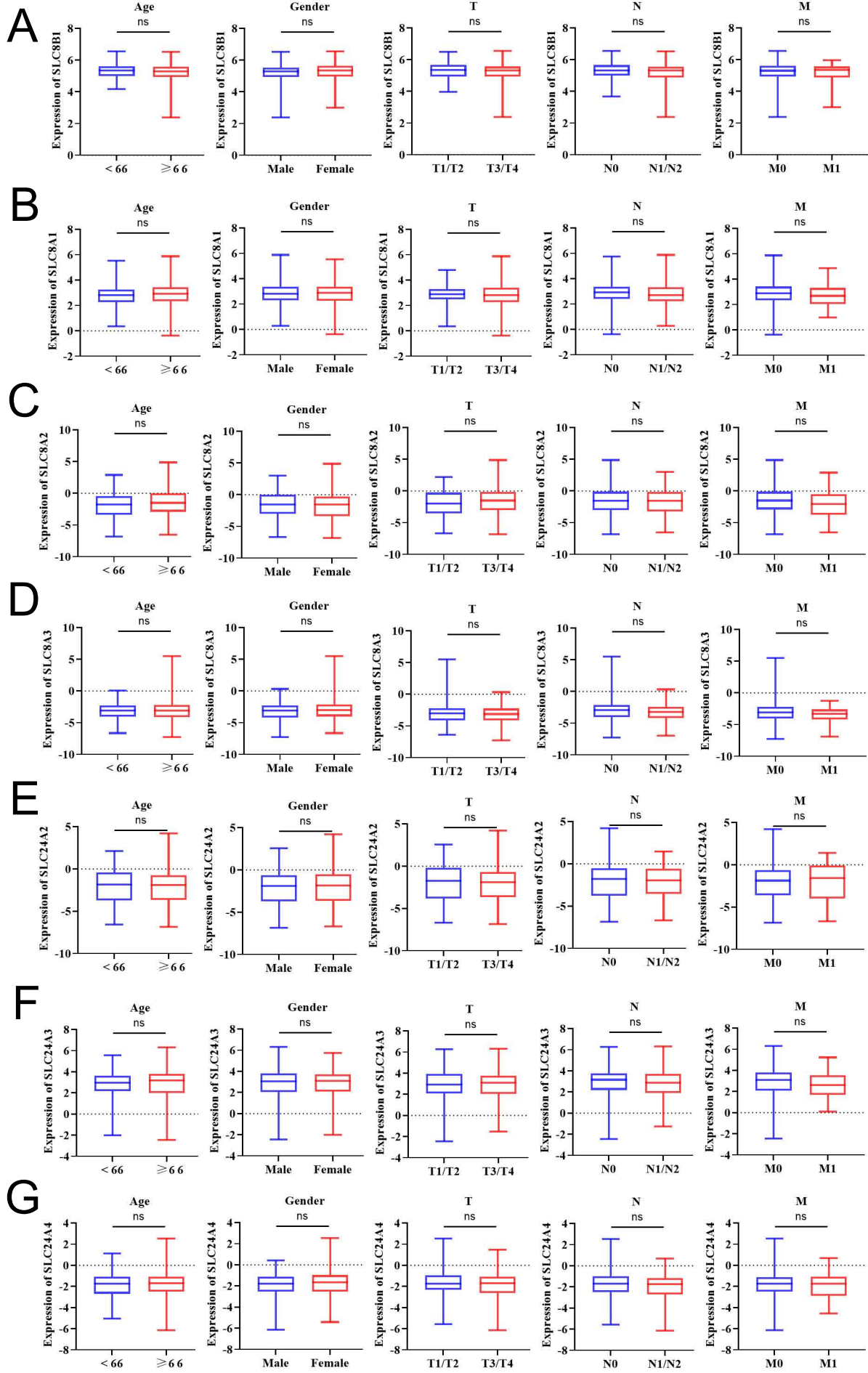

Supplement: Figure S2 [file peerj-12-17582-s002.pdf]

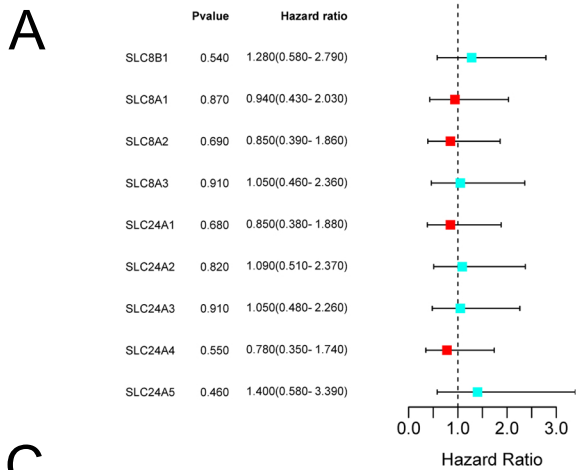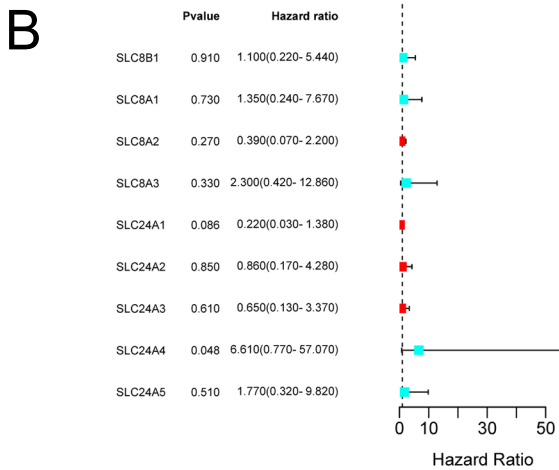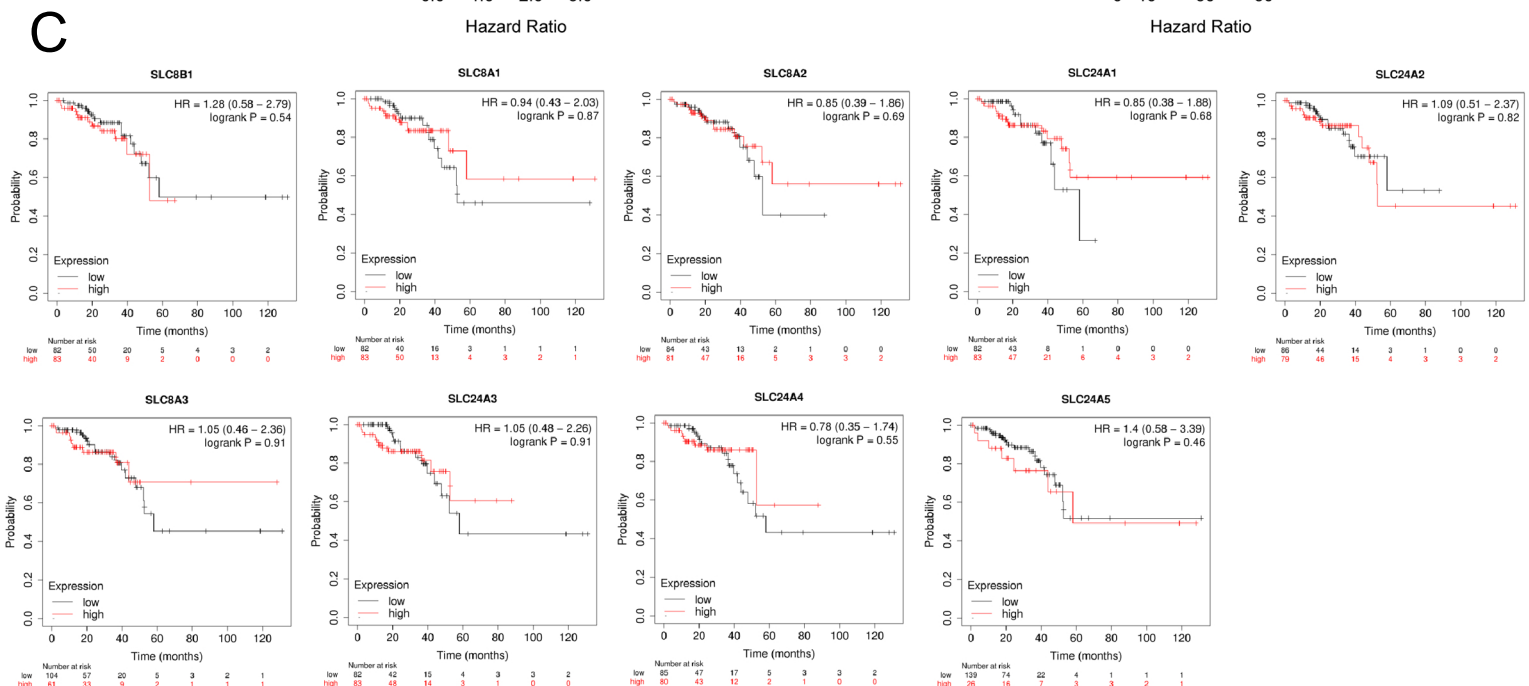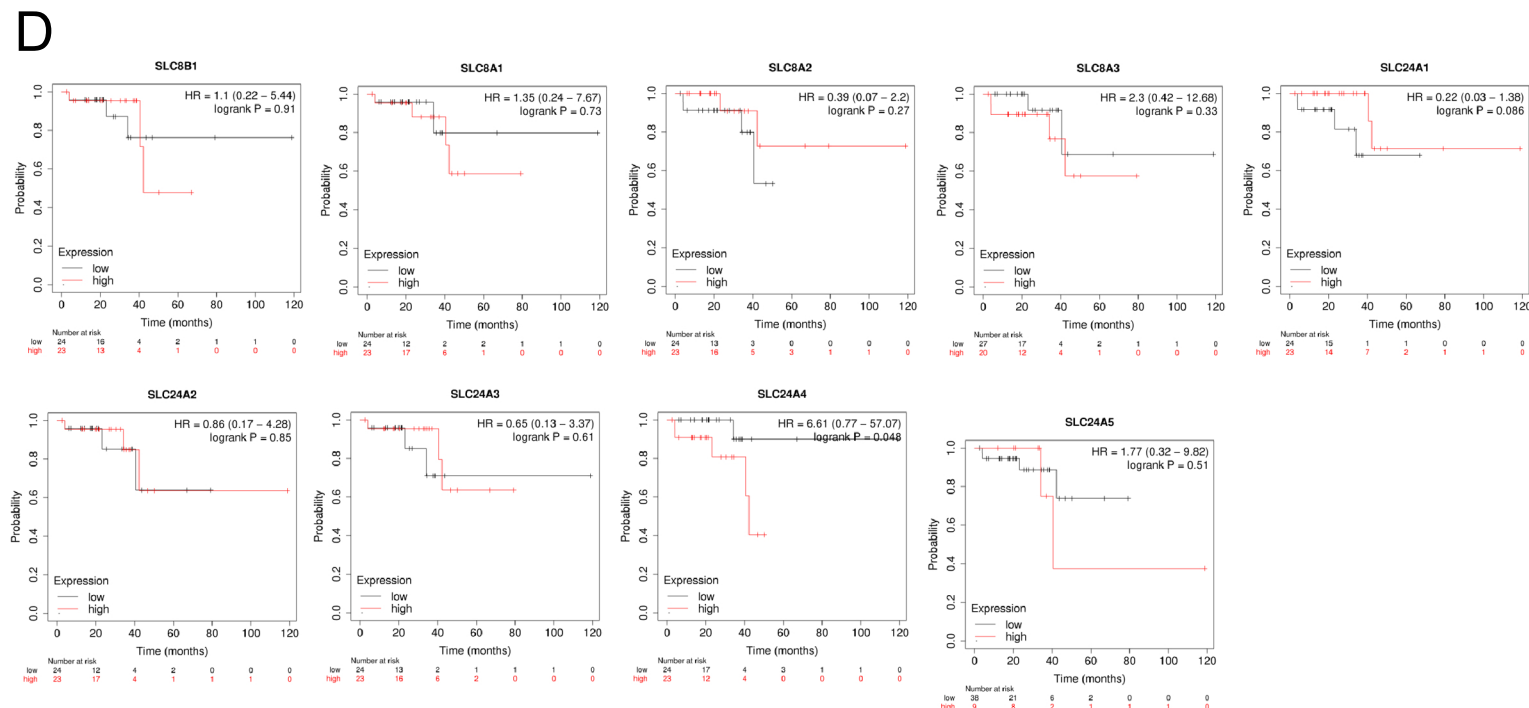

Supplement: Figure S3 — (A) overall survival (OS) analyses. (B) disease-free survival (DFS) analyses. (C) Kaplan–Meier Plotter. [file peerj-12-17582-s003.pdf]

A

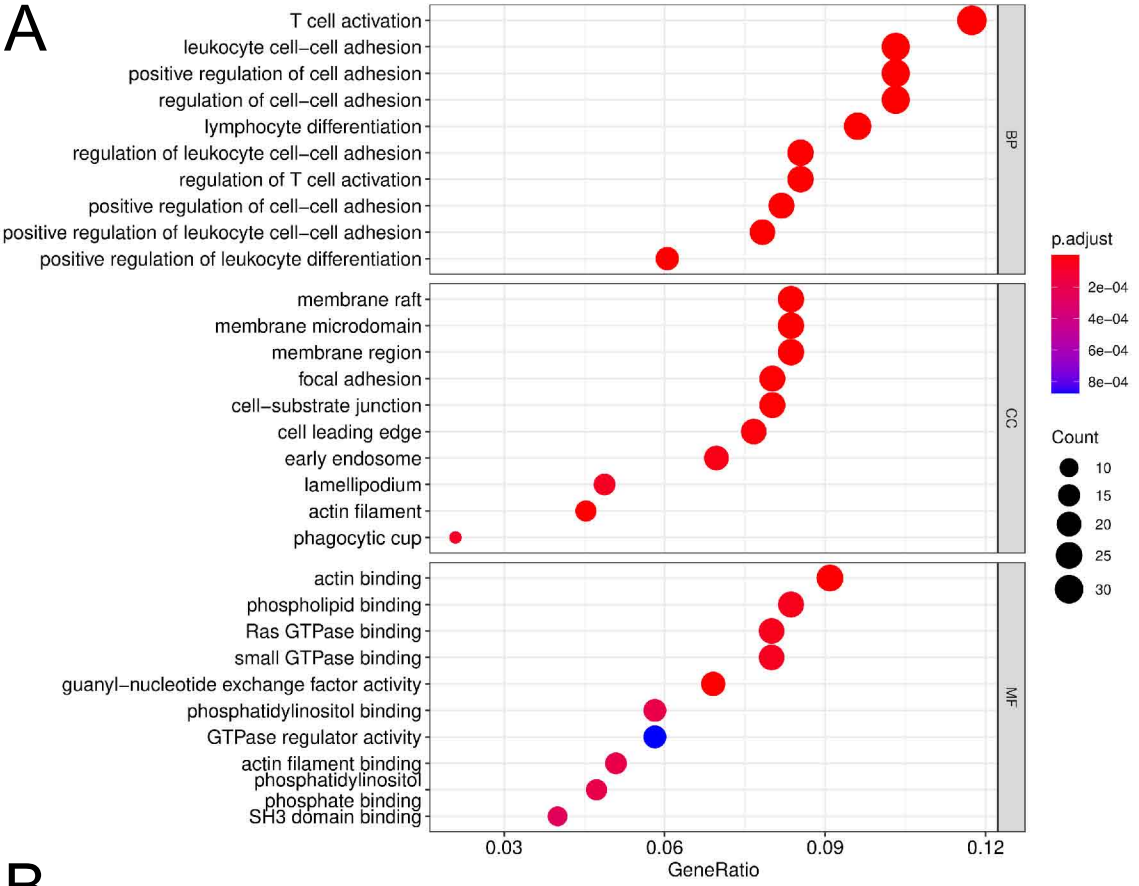

B

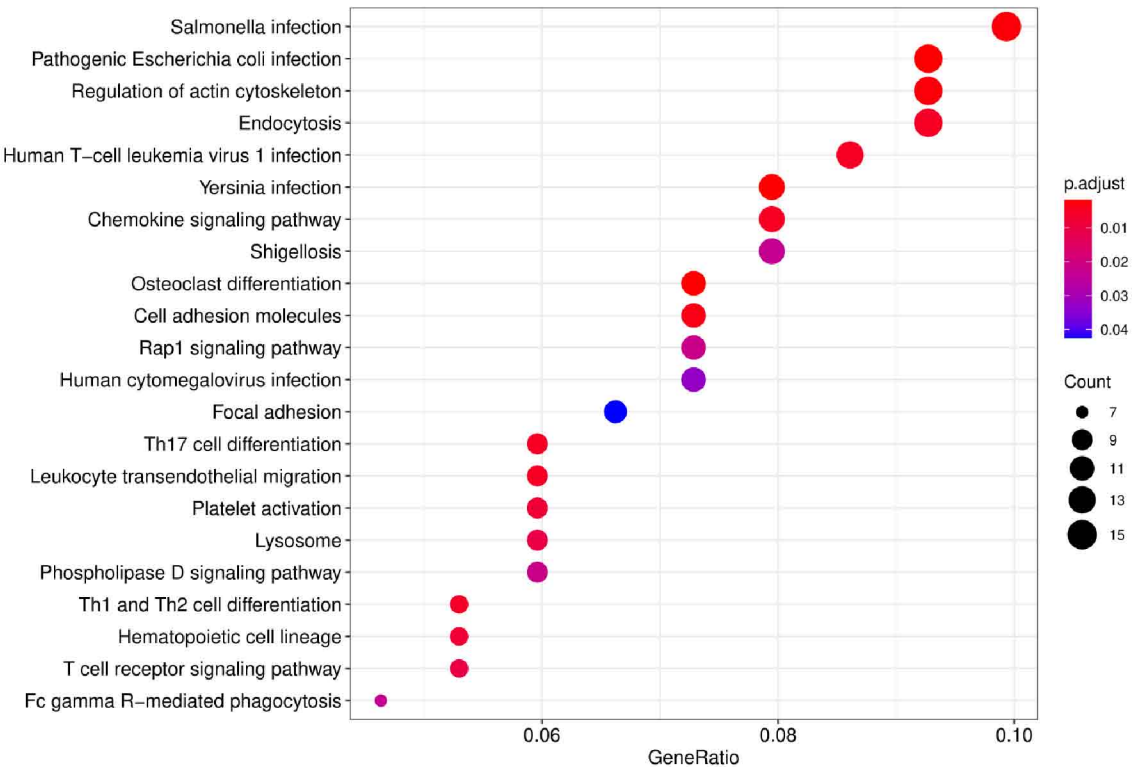

Supplement: Figure S6 — (A) Gene Ontology (GO) analysis. (B) Kyoto Encyclopedia of Genes and Genomes (KEGG) analysis. [file peerj-12-17582-s006.pdf]

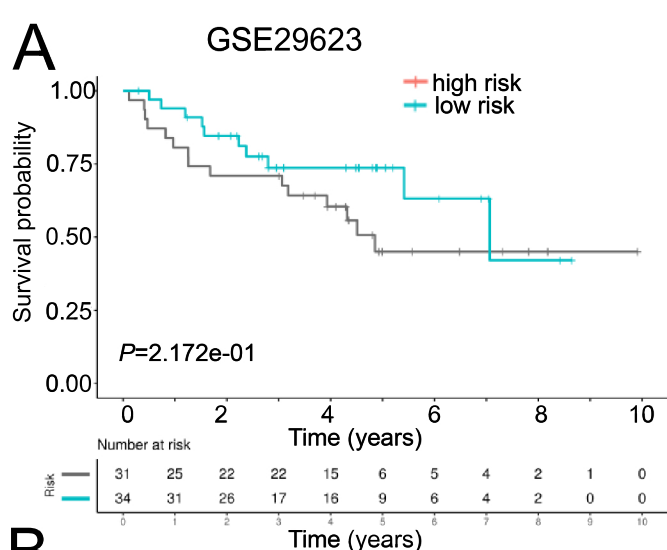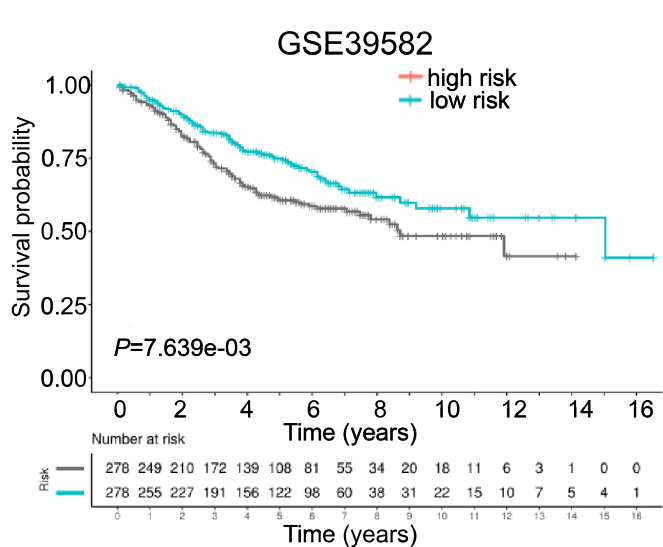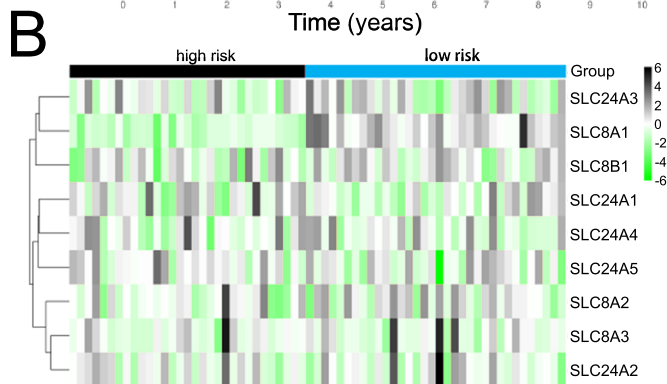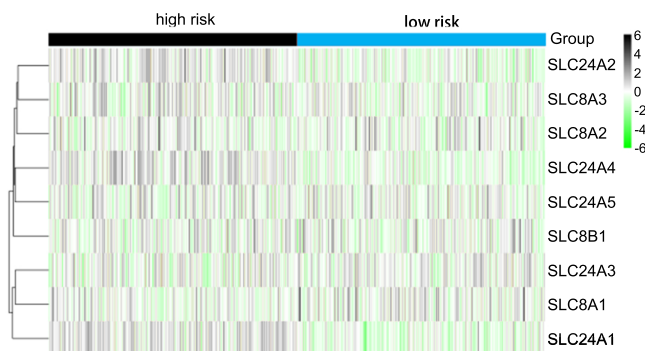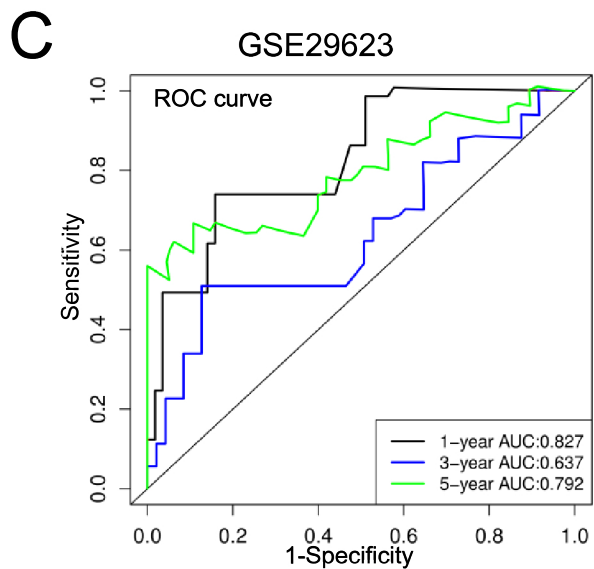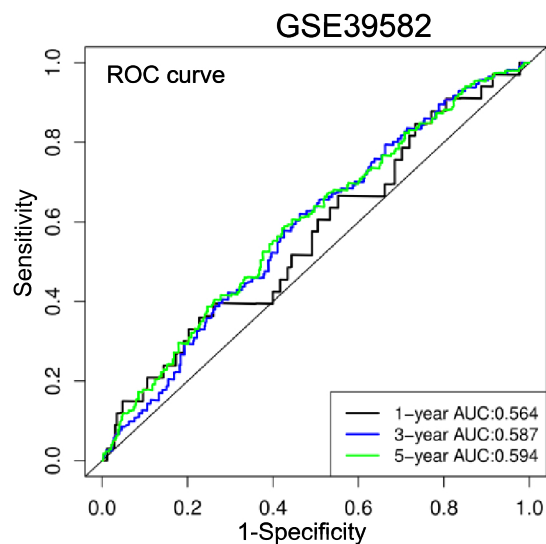

Supplement: Figure S7 — (A) K–M survival between low-risk and high-risk group. (B) The gene expression of calcium extrusion-related genes in GSE29623 and GSE39582. (C) ROC curve and AUC at 1, 3, and 5 years for the risk score in GSE29623 and GSE39582. * P < 0.05; ** P < 0.01; *** P < 0.001. [file peerj-12-17582-s007.pdf]

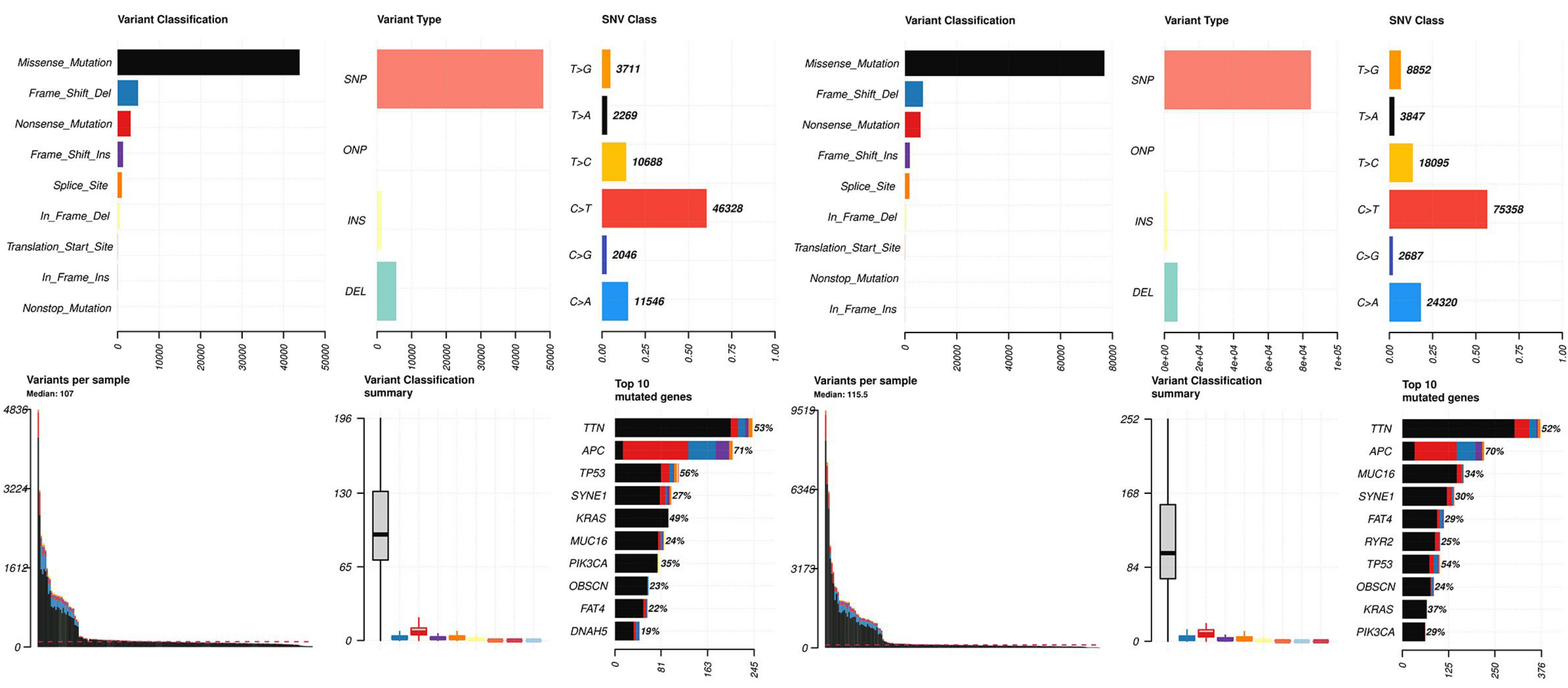

Supplement: Figure S8 [file peerj-12-17582-s008.pdf]

A

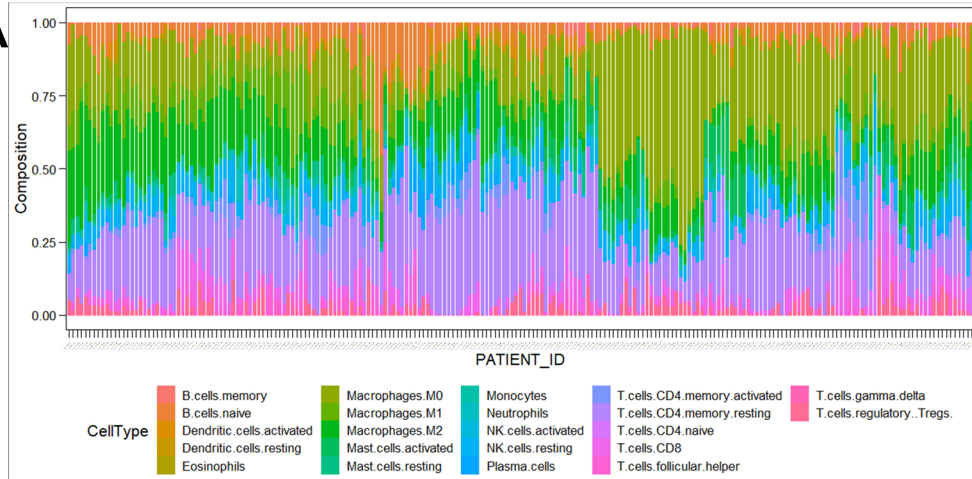

B

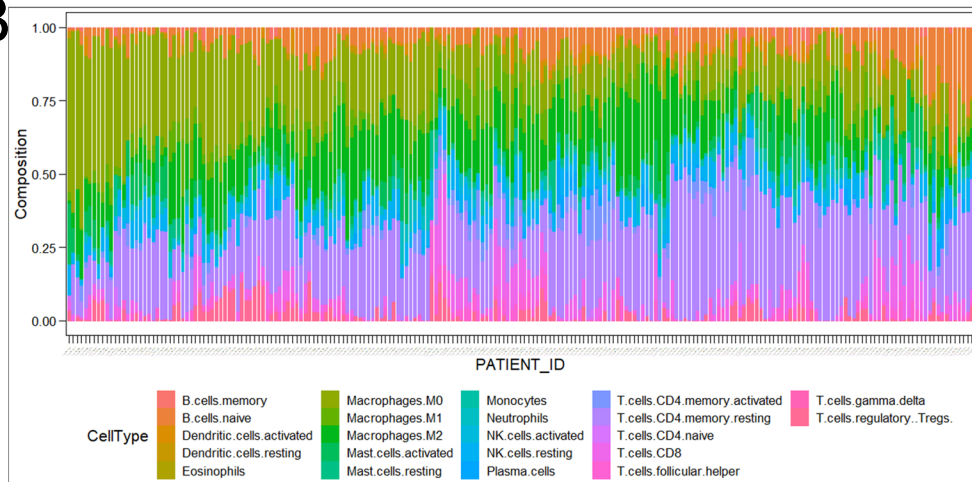

D

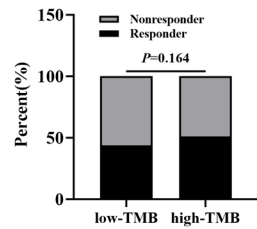

E

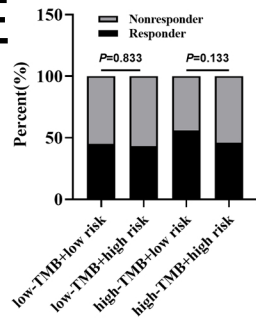

F

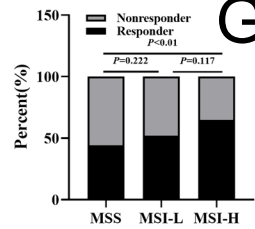

G

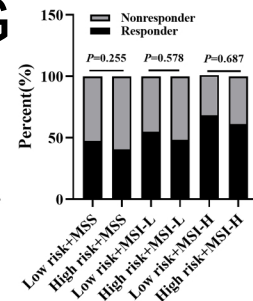

C

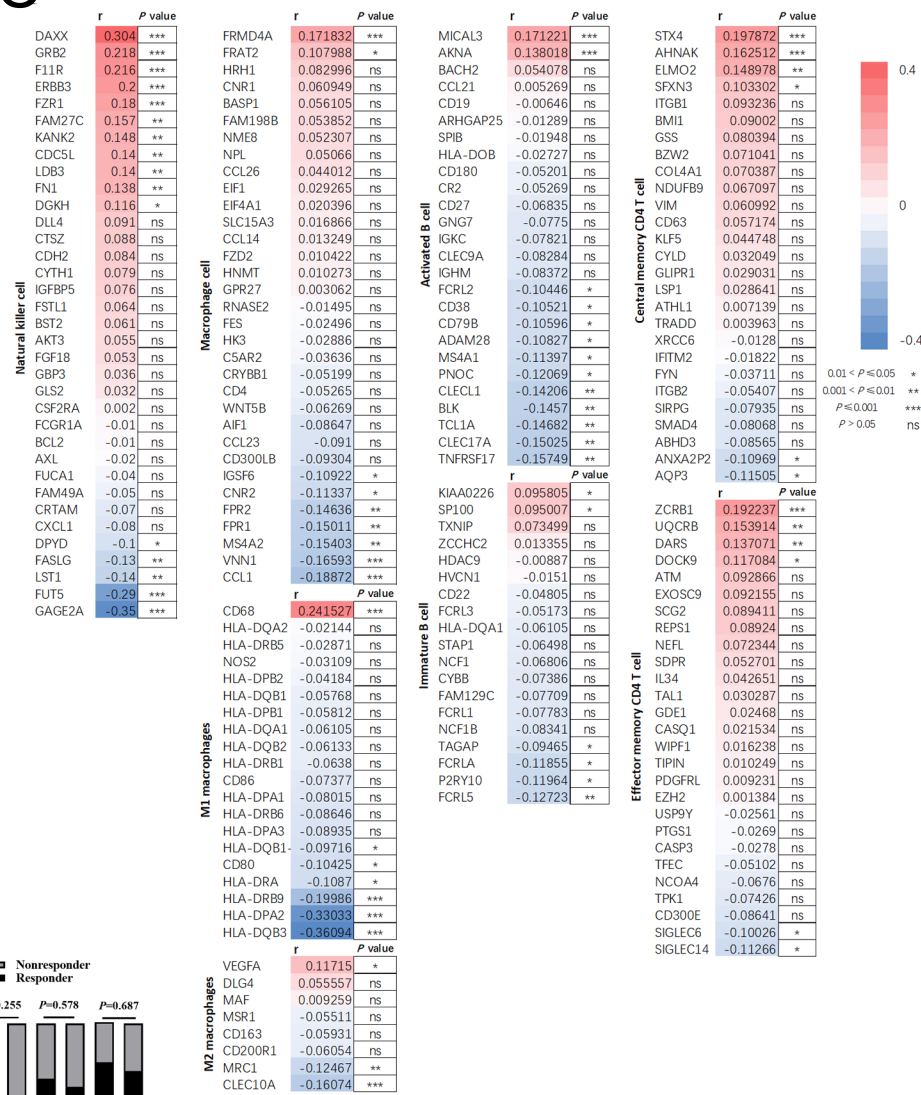

Supplement: Figure S9 — (A) The abundance of 22 immune infiltrates between the low-risk and high-risk group. (B) The relationship between risk score and the expression of immune cell marker genes (D) TIDE predicted the proportion of patients with response to immunotherapy in low-TMB and high-TMB groups. (E) TIDE predicted the proportion of patients of four groups based on the risk score and TMB level with response to immunotherapy. (F) TIDE predicted the proportion of patients with response to immunotherapy in MSS, MSI-L and MSI-H groups. (G) TIDE predicted the proportion of patients of six groups based on the risk score and microsatellite status with response to immunotherapy. MSS, Microsatellite stability; MSI-L, Microsatellite Instability-Low; MSI-H, Microsatellite Instability-High. [file peerj-12-17582-s009.pdf]

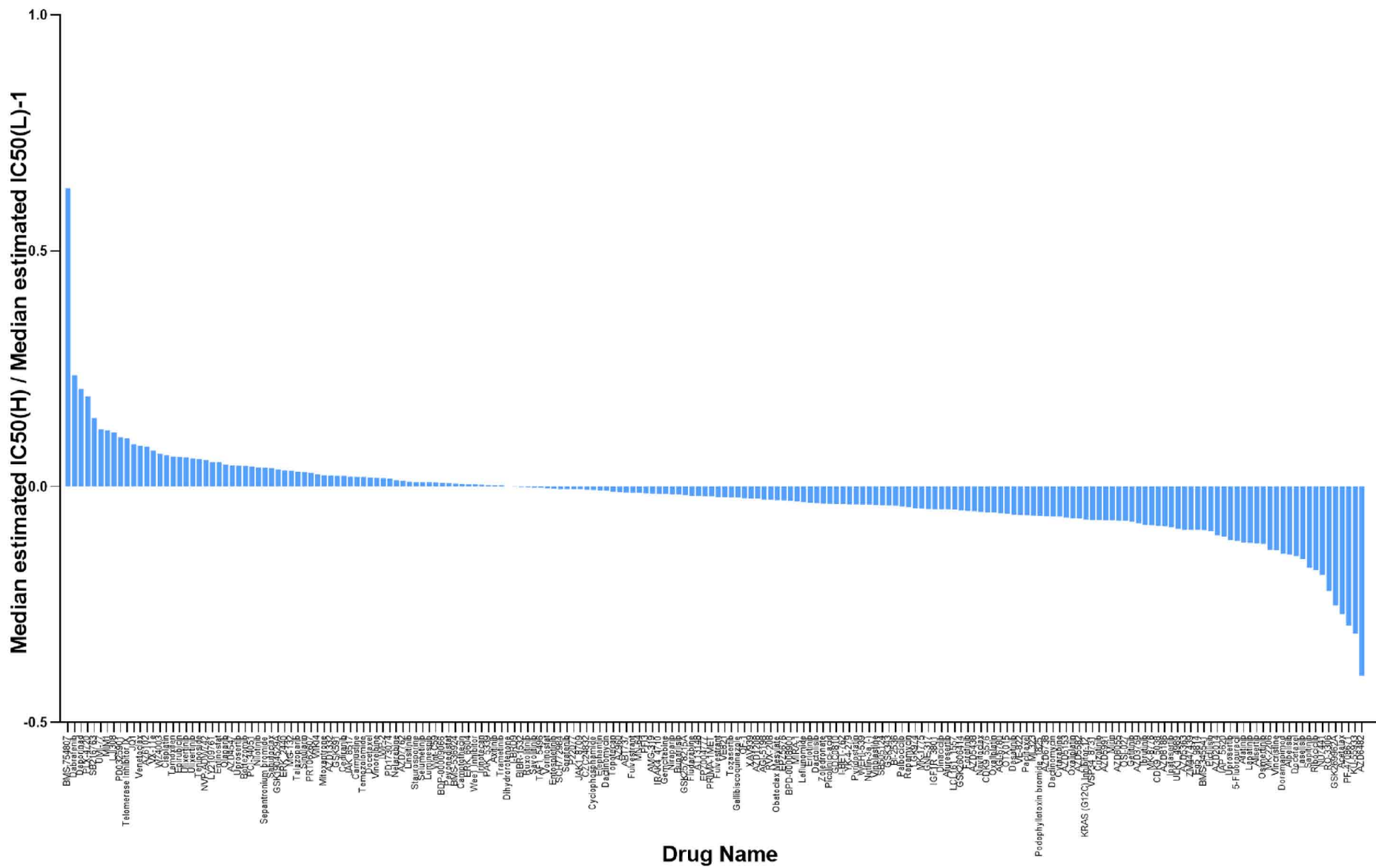

Supplement: Figure S10 [file peerj-12-17582-s010.pdf]

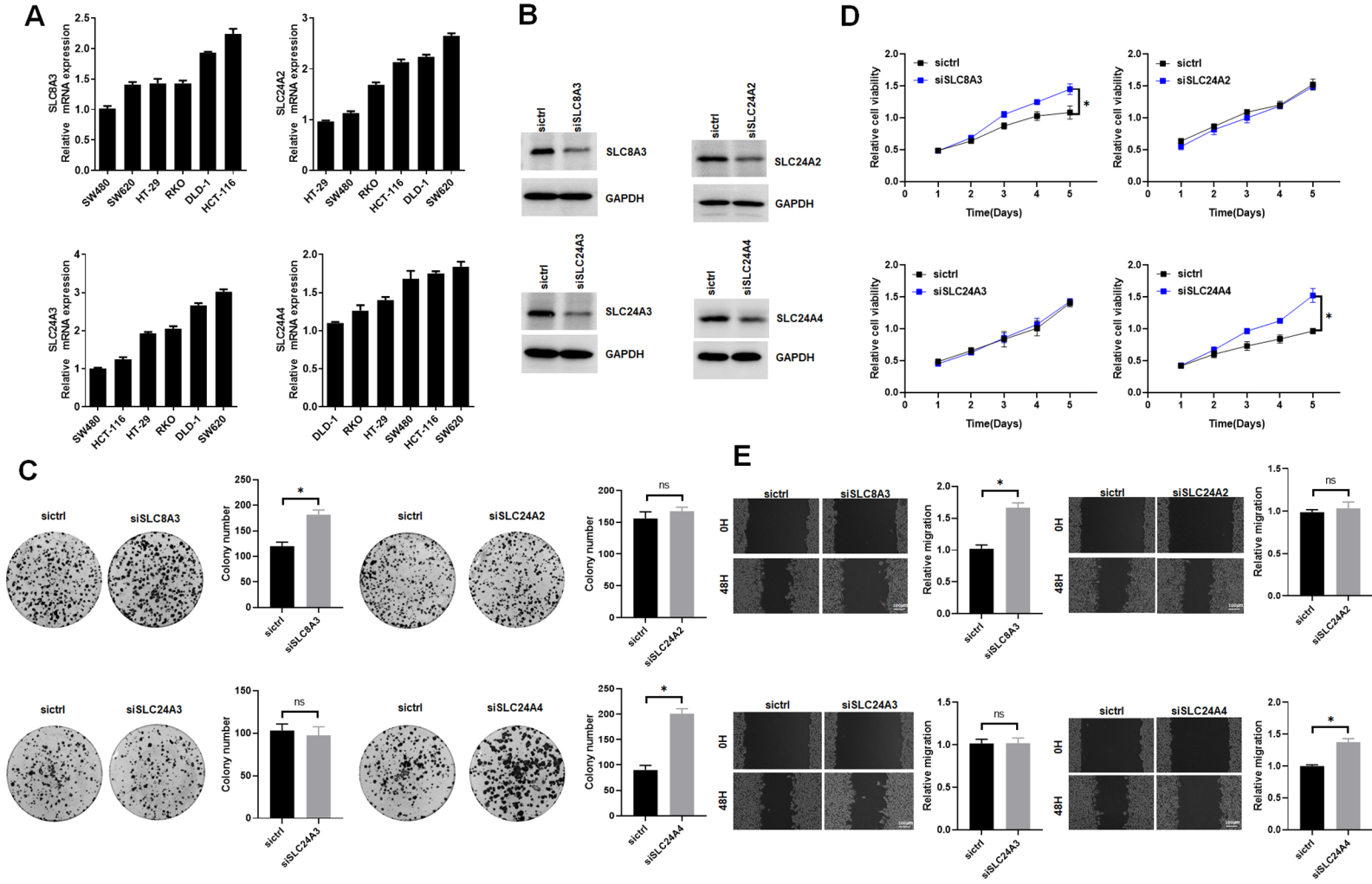

Supplement: Figure S11 — (A) the mRNA expression level of SLC8A3, SLC24A2, SLC24A3 and SLC24A4 in several CRC cell lines (B)Western blotting analysis to measure SLC8A3, SLC24A2, SLC24A3 and SLC24A4 protein expression in RKO cells, treated as indicated. (C) CCK8 assays (D)Scratch wound healing (E) colony formation assay of RKO cells, treated as indicated. [file peerj-12-17582-s011.pdf]
