# Supplementary material for: Identification and validation of calcium extrusion-related genes prognostic signature in colon adenocarcinoma
Source: PeerJ. 2024 Jul 10;12:e17582. doi: 10.7717/peerj.17582 (PMC11246022; doi:10.7717/peerj.17582)
Supplement: Figure S4 — (A) Overview the genetic variations analyses of calcium extrusion-related genes. (B) Correlation analysis between the mRNA expression of calcium extrusion-related genes and TMB of COAD patients. (C) Protein-protein interaction network analysis through STRING database. (D) Correlation analysis of the calcium extrusion-related genes. (E) Gene-gene interaction network analysis through GeneMANIA. [file peerj-12-17582-s004.pdf]

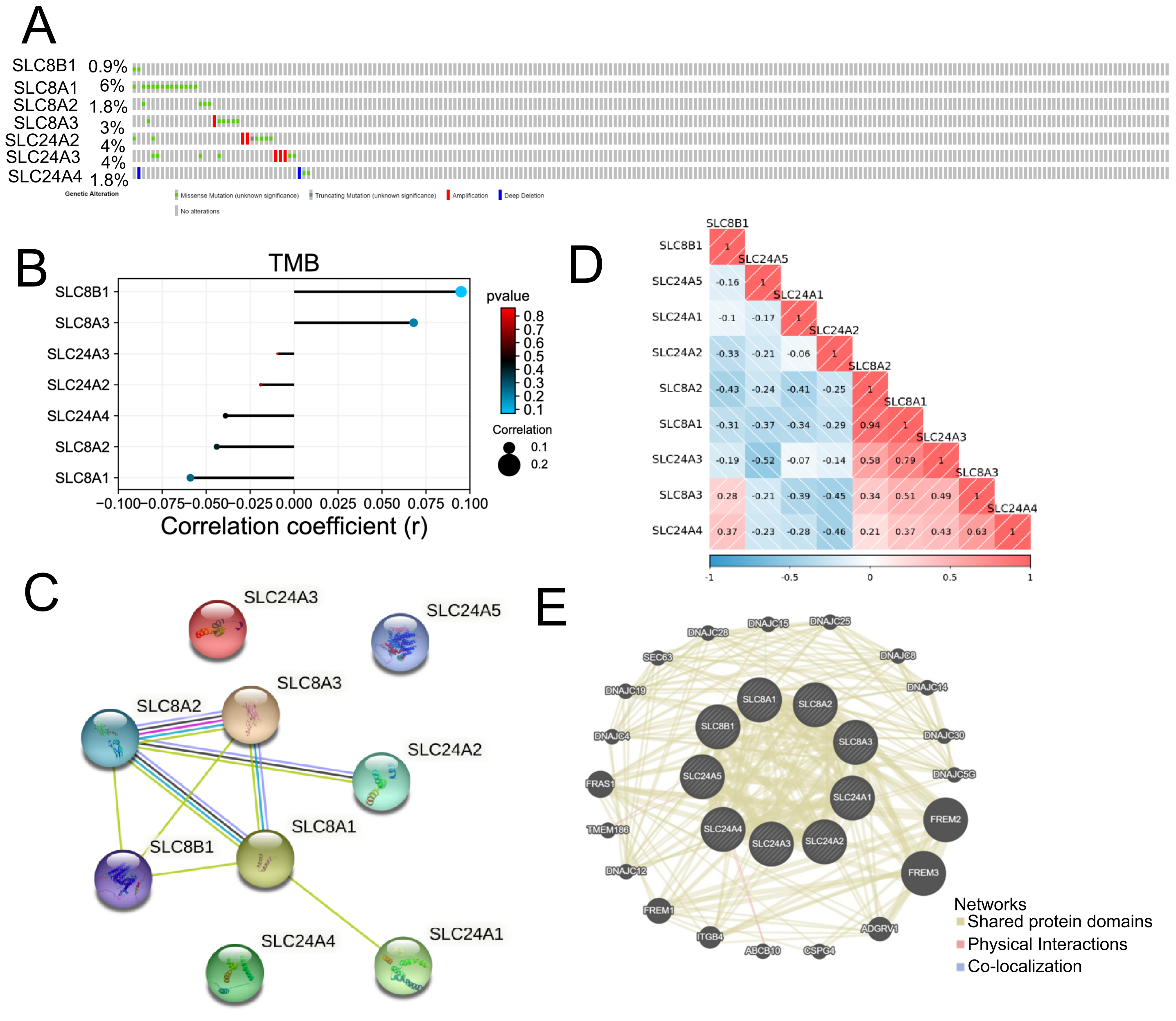
A
SLC8B1 0.9%
SLC8A1 6%
SLC8A2 1.8%
SLC8A3 3%
SLC24A2 4%
SLC24A3 4%
SLC24A4 1.8%
Genetic Alteration
Missense Mutation (unknown significance)
Truncating Mutation (unknown significance)
Amplification
Deep Deletion
No alterations
B
TMB
pvalue
Correlation
Correlation coefficient (r)
C
D
E
Networks
Shared protein domains
Physical Interactions
Co-localization
