# Supplementary material for: Identification and validation of calcium extrusion-related genes prognostic signature in colon adenocarcinoma
Source: PeerJ. 2024 Jul 10;12:e17582. doi: 10.7717/peerj.17582 (PMC11246022; doi:10.7717/peerj.17582)
Supplement: Table S1 [file peerj-12-17582-s012.docx]

**Supplementary Table S1** Detail information of nine calcium extrusion-related genes

| Gene symbol | Full name | Function of the encoded protein | Subcellular Location |
| --- | --- | --- | --- |
| SLC8B1 | solute carrier family 8 member B1 | SLC8B1 encodes a member of potassium-dependent sodium/calcium exchangers that maintain cellular calcium homeostasis through the electrogenic countertransport of 4 sodium ions for 1 calcium ion and 1 potassium ion |  |
| SLC8A1 | solute carrier family 8 member A1 | The Na^+^-Ca^2+^ exchanger is the primary mechanism by which the Ca^2+^ is extruded from the cell during relaxation. | Nucleoplasm;  Plasma membrane |
| SLC8A2 | solute carrier family 8 member A2 | Predicted to enable calcium:cation antiporter activity involved in regulation of postsynaptic cytosolic calcium ion concentration and calcium:sodium antiporter activity. |  |
| SLC8A3 | solute carrier family 8 member A3 | This gene encodes a member of the sodium/calcium exchanger integral membrane protein family |  |
| SLC24A1 | solute carrier family 24 member 1 | This gene encodes a member of the potassium-dependent sodium/calcium exchanger protein family mediating the extrusion of one calcium ion and one potassium ion in exchange for four sodium ions | Microtubules |
| SLC24A2 | Solute carrier family 24 member 2 | This gene encodes a member of the calcium/cation antiporter superfamily of transport proteins which can mediate the extrusion of one Ca^2+^ ion and one K^+^ ion in exchange for four Na^+^ ions. | Plasma membrane |
| SLC24A3 | solute carrier family 24 member 3 | Potassium-dependent sodium/calcium exchangers are believed to transport 1 intracellular calcium and 1 potassium ion in exchange for 4 extracellular sodium ions | Golgi apparatus; Cytosol |
| SLC24A4 | solute carrier family 24 member 4 | This gene encodes a member of the potassium-dependent sodium/calcium exchanger protein family. | Plasma membrane |
| SLC24A5 | solute carrier family 24 member 5 | This gene is a member of the potassium-dependent sodium/calcium exchanger family |  |
