# Supplementary material for: Identification and validation of calcium extrusion-related genes prognostic signature in colon adenocarcinoma
Source: PeerJ. 2024 Jul 10;12:e17582. doi: 10.7717/peerj.17582 (PMC11246022; doi:10.7717/peerj.17582)
Supplement: Table S2 [file peerj-12-17582-s013.docx]

Table S2. Clinical and pathological data of patients included in TCGA, GSE39582, GSE29623 and GSE17536.

| Characteristic | TCGA (n, %) | GSE39582(n, %) | GSE29623(n, %) | GSE17536(n, %) |
| --- | --- | --- | --- | --- |
| Samples | **471(100%)** | **575(100.00%)** | **65(100.00%)** | **177(100.00%)** |
| Normal | 41(8.7%) | 19(3.30%) | 0 | 0 |
| Tumor | 430(91.3%) | 556(96.70%) | 65(100.00%) | 177(100.00%) |
| Survival Status | **430(100.00%)** | **556(100.00%)** | **65(100.00%)** | **177(100.00%)** |
| Death | 94(21.86%) | 187(33.63%) | 25(38.46%) | 73(41.24%) |
| Survival | 336(78.14%) | 369(66.37%) | 40(61.54%) | 104(58.76%) |
| Age | **430(100.00%)** | **555(99.82%)** | **NA** | **177(100.00%)** |
| ≤66 | 192(44.65%) | 236(42.45%) |  | 91(51.43%) |
| >66 | 238(55.35%) | 319(57.37%) |  | 86(48.59%) |
| Gender | **430(100.00%)** | **556(100.00%)** | **65(100.00%)** | **177(100.00%)** |
| Female | 198(46.05%) | 249(44.78%) | 25(38.46%) | 81(45.76%) |
| Male | 232(53.95%) | 307(55.22%) | 40(61.54%) | 96(54.24%) |
| Stage | **419(97.44%)** | **552(99.28%)** | **NA** | **NA** |
| Ⅰ | 73(16.98%) | 32(5.76%) |  |  |
| Ⅱ | 165(38.37%) | 258(46.40%) |  |  |
| Ⅲ | 121(28.14%) | 203(36.51%) |  |  |
| Ⅳ | 60(13.95%) | 59(10.61%) |  |  |
| T classification | **429(99.77%)** | **532(95.68%)** | **65(100.00%)** | **NA** |
| T1 | 11(2.56%) | 11(1.98%) | 0 |  |
| T2 | 75(17.44%) | 44(7.91%) | 8(12.31%) |  |
| T3 | 294(68.37%) | 360(64.75%) | 52(80.00%) |  |
| T4 | 49(11.40%) | 117(21.04%) | 5(7.69%) |  |
| N classification | **430(100.00%)** | **530(95.32%)** | **64(98.5%)** | **NA** |
| N0 | 253(58.84%) | 295(53.05%) | 32(49.23%) |  |
| N1 | 100(23.26%) | 131(23.56%) | 25(38.46%) |  |
| N2 | 77(17.90%) | 98(17.63%) | 7(10.77%) |  |
| N3 | 0 | 6(1.08%) | 0 |  |
| M classification | **378(87.90%)** | **534(96.04%)** | **64(98.5%)** | **NA** |
| M0 | 318(73.95%) | 474(85.25%) | 46(70.77%) |  |
| M1 | 60(13.95%) | 60(10.79%) | 18(27.69%) |  |
