# Supplementary material for: Identification and validation of calcium extrusion-related genes prognostic signature in colon adenocarcinoma
Source: PeerJ. 2024 Jul 10;12:e17582. doi: 10.7717/peerj.17582 (PMC11246022; doi:10.7717/peerj.17582)
Supplement: Supplemental Information 2 [file peerj-12-17582-s015.pdf]

Figure 9A

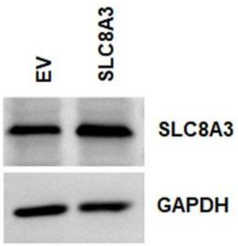
EV
SLC8A3
SLC8A3
GAPDH

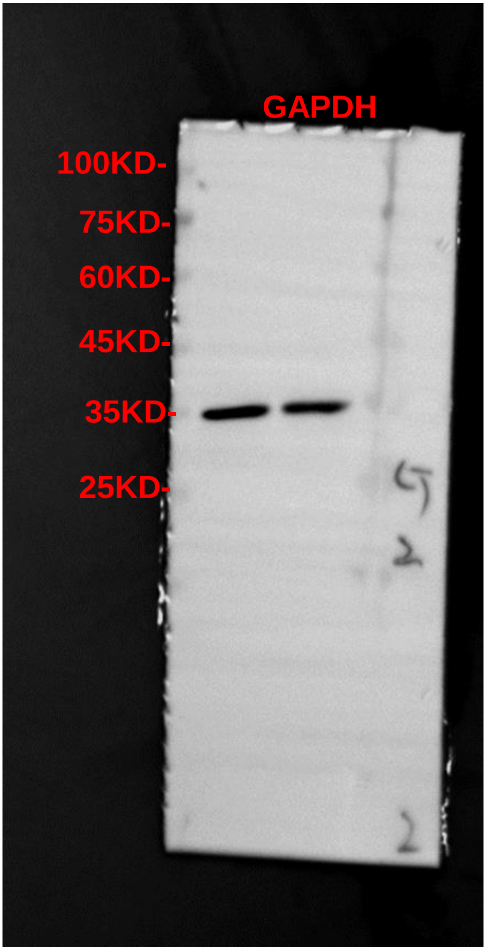
GAPDH
100KD-
75KD-
60KD-
45KD-
35KD-
25KD-

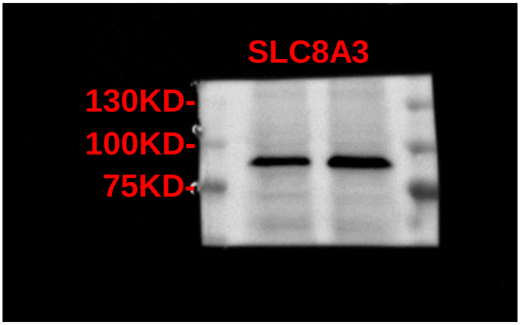
SLC8A3
130KD-
100KD-
75KD-

Figure 9A

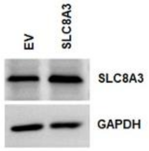

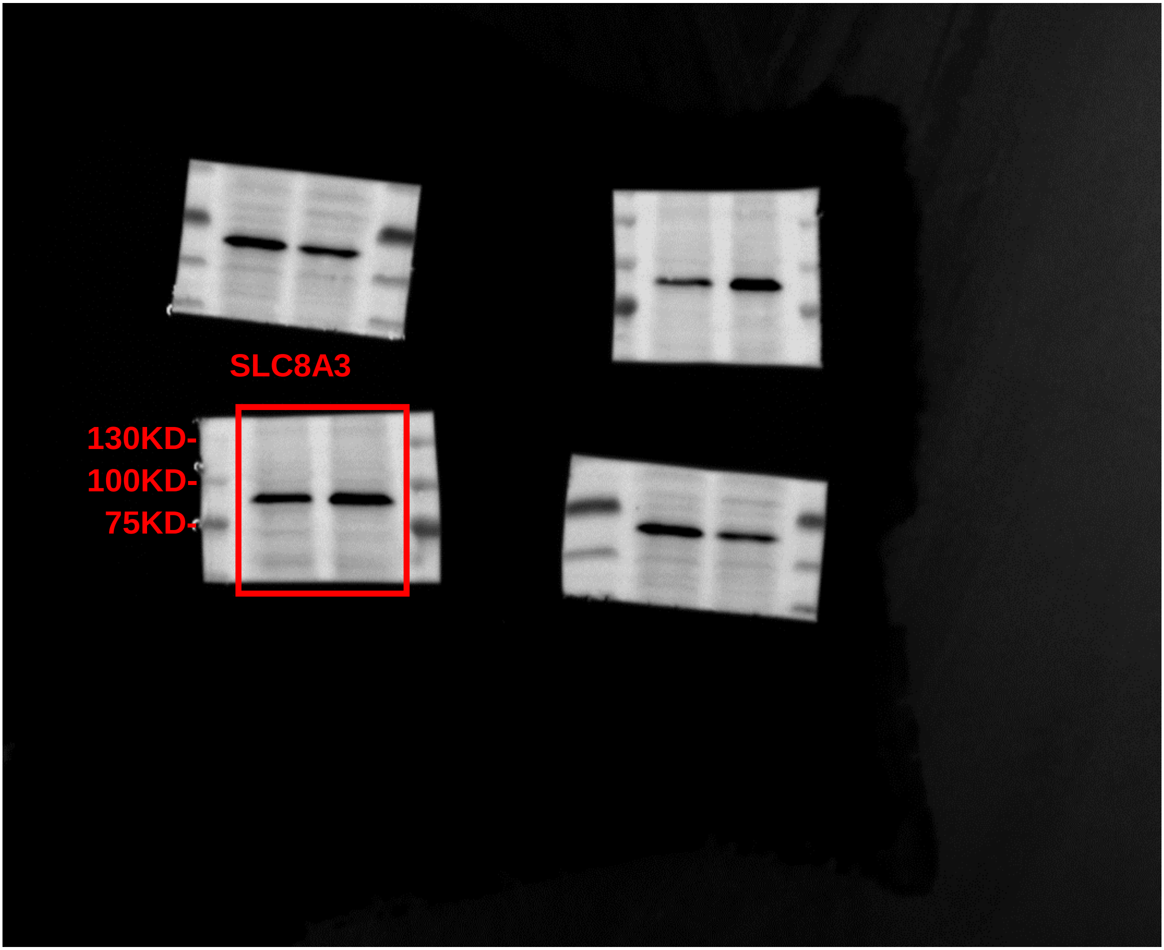

Figure 9A

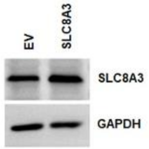

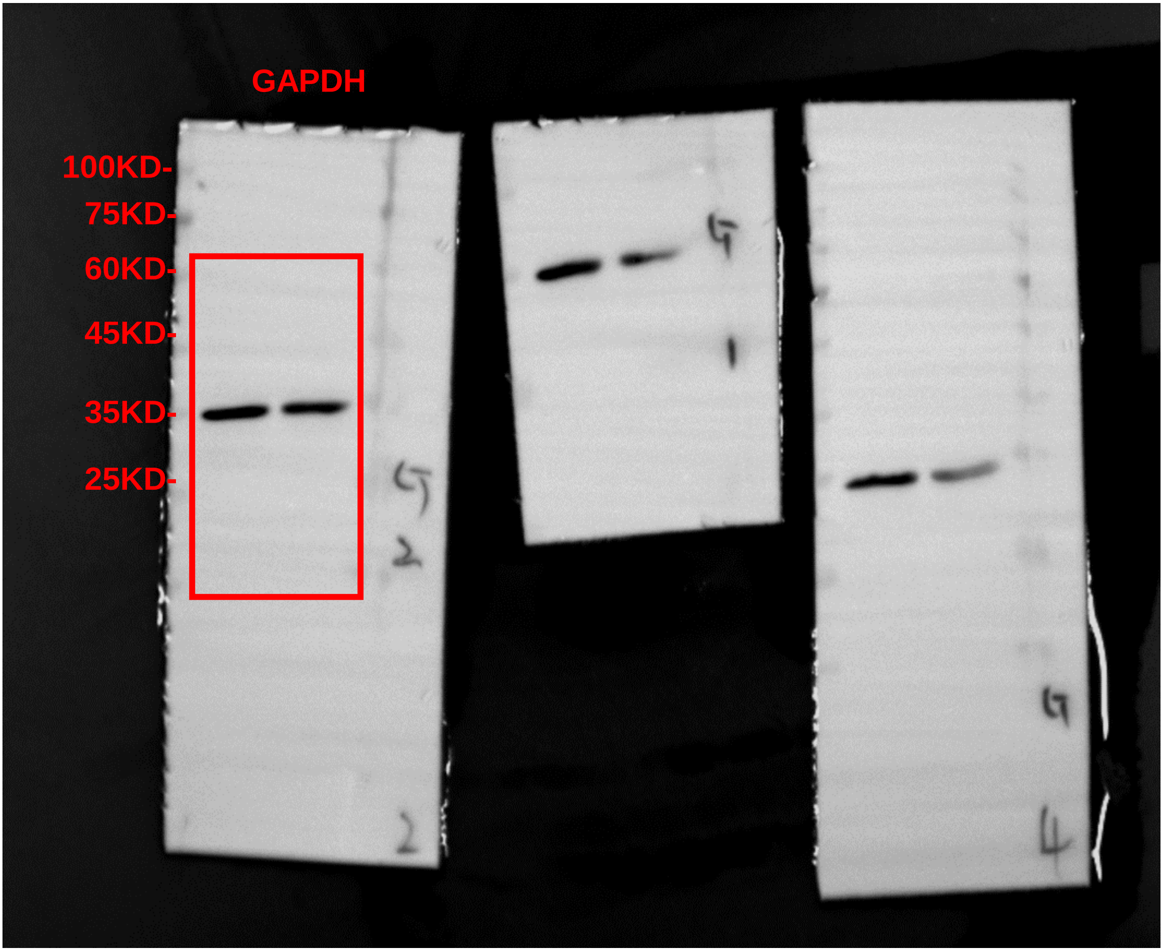

Figure 9A

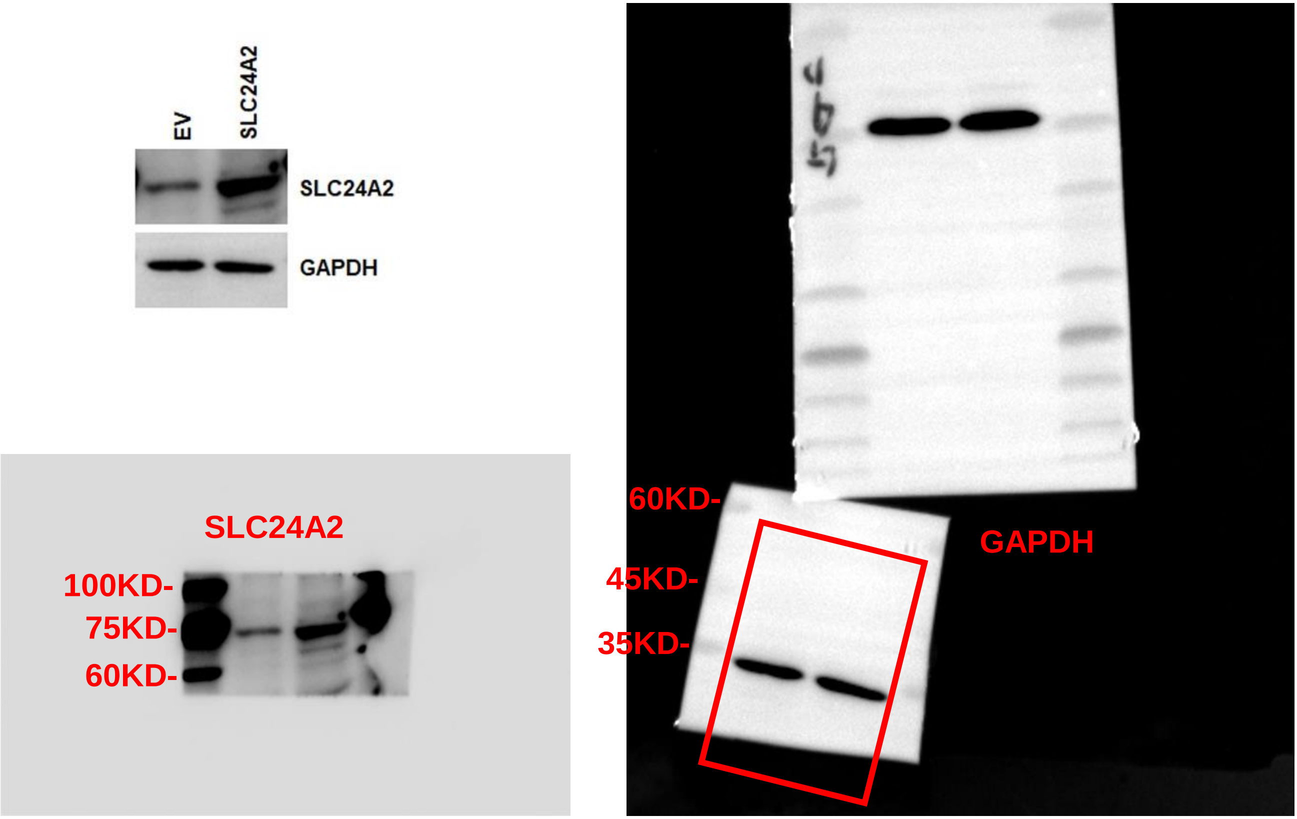
EV
SLC24A2
SLC24A2
GAPDH
SLC24A2
100KD-
75KD-
60KD-
60KD-
45KD-
35KD-
GAPDH

Figure 9A

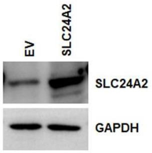

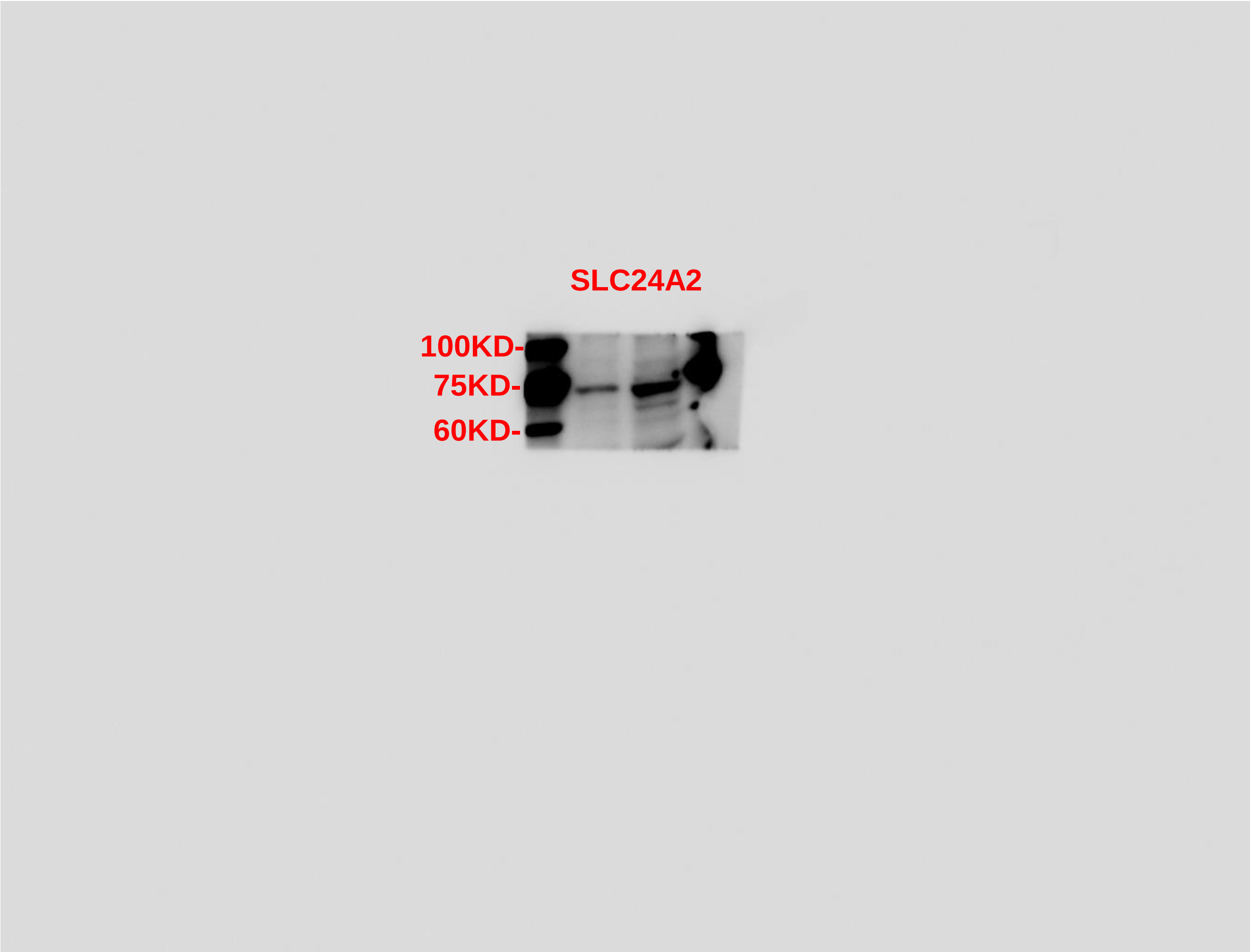

Figure 9A

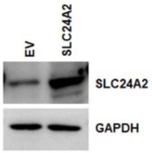

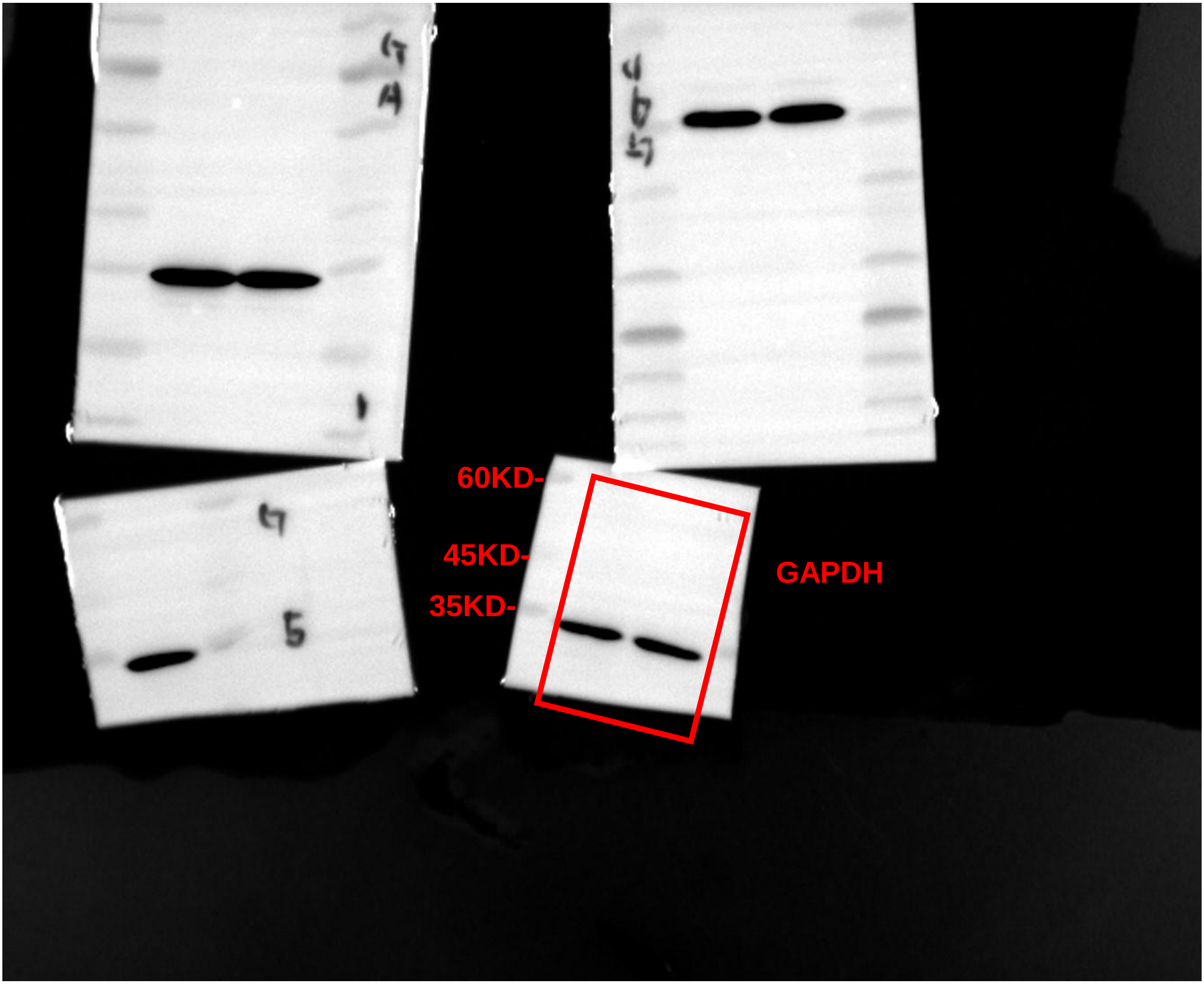

Figure 9A

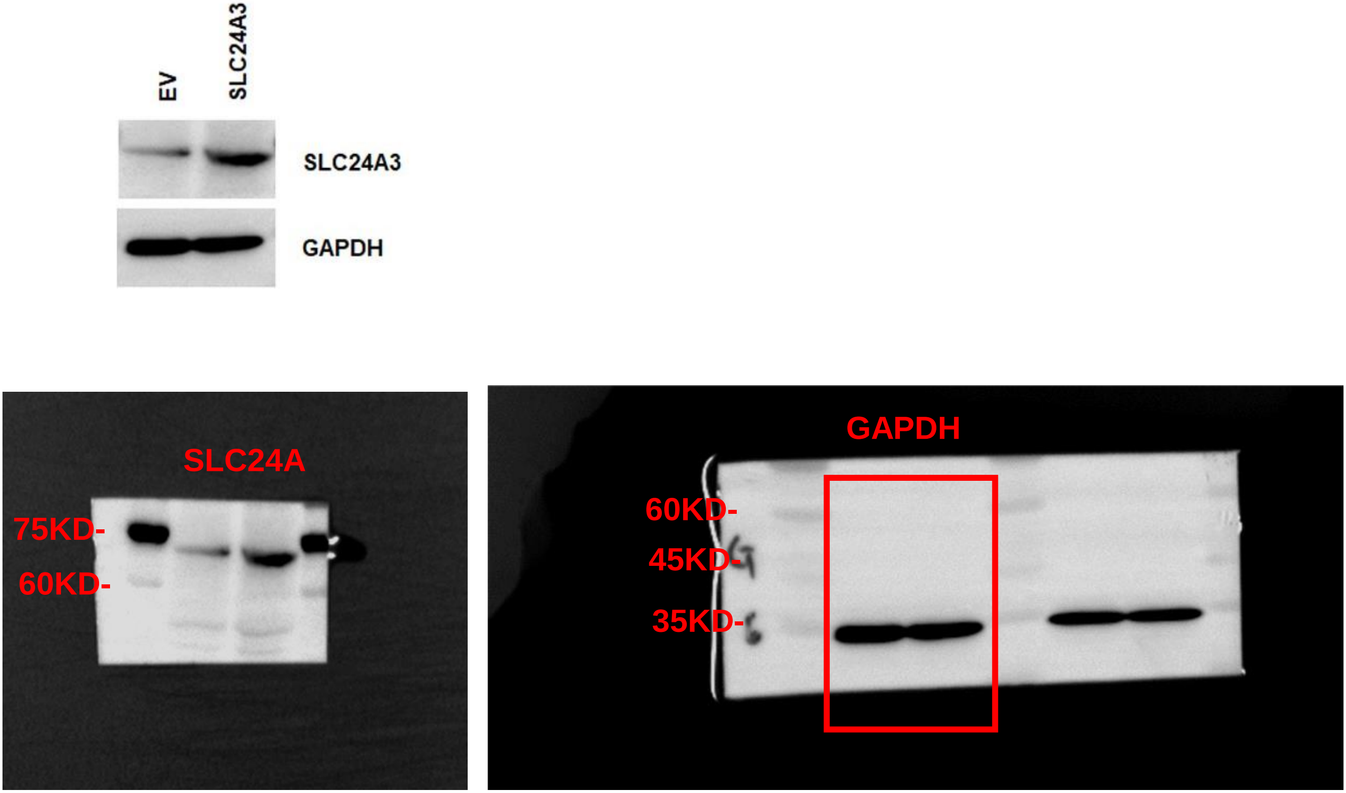
EV
SLC24A3
SLC24A3
GAPDH
SLC24A
75KD-
60KD-
GAPDH
60KD-
45KD-
35KD-

Figure 9A

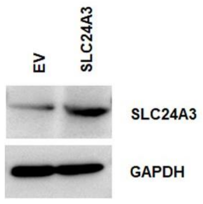

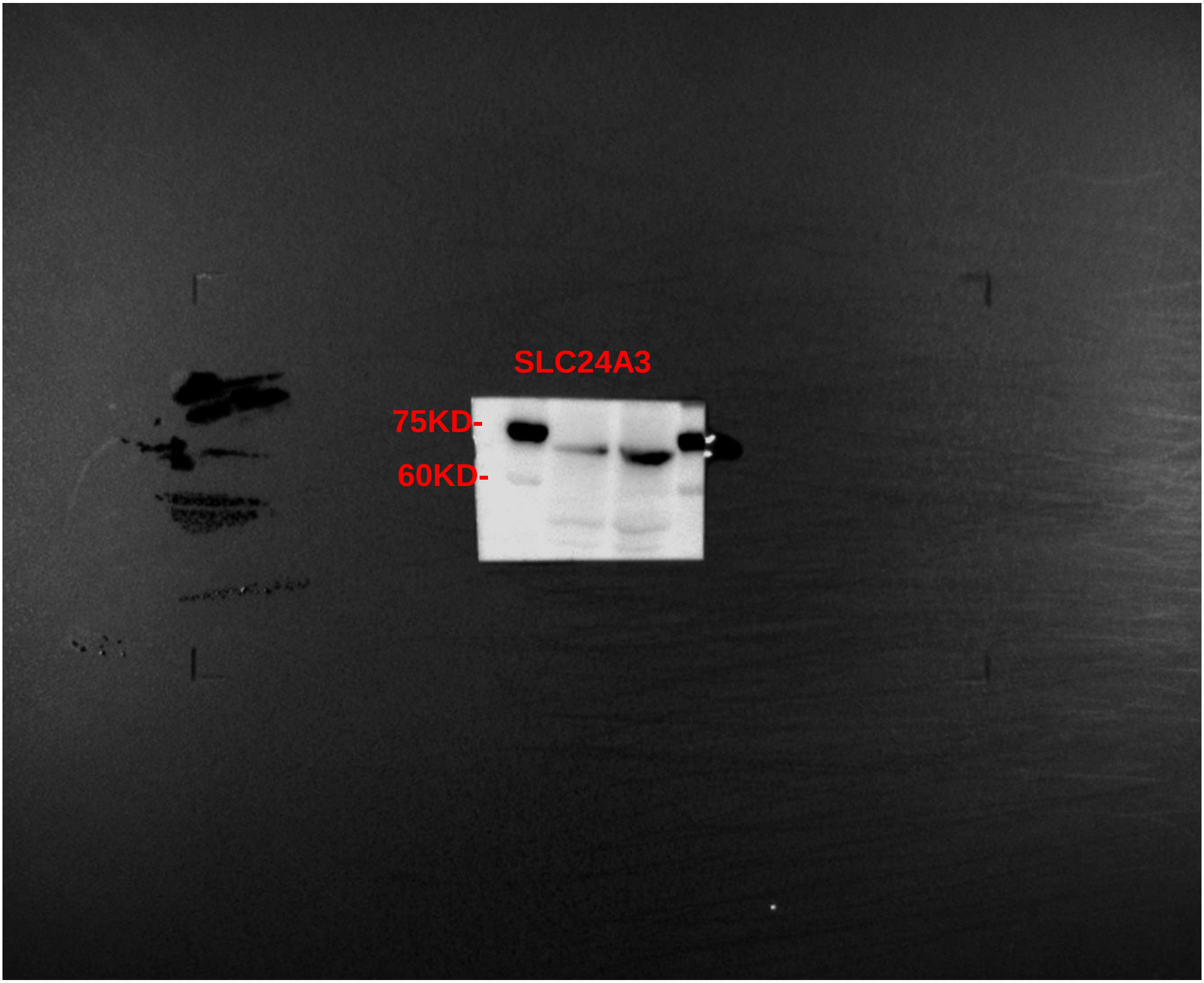

Figure 9A

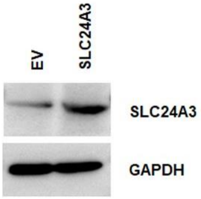

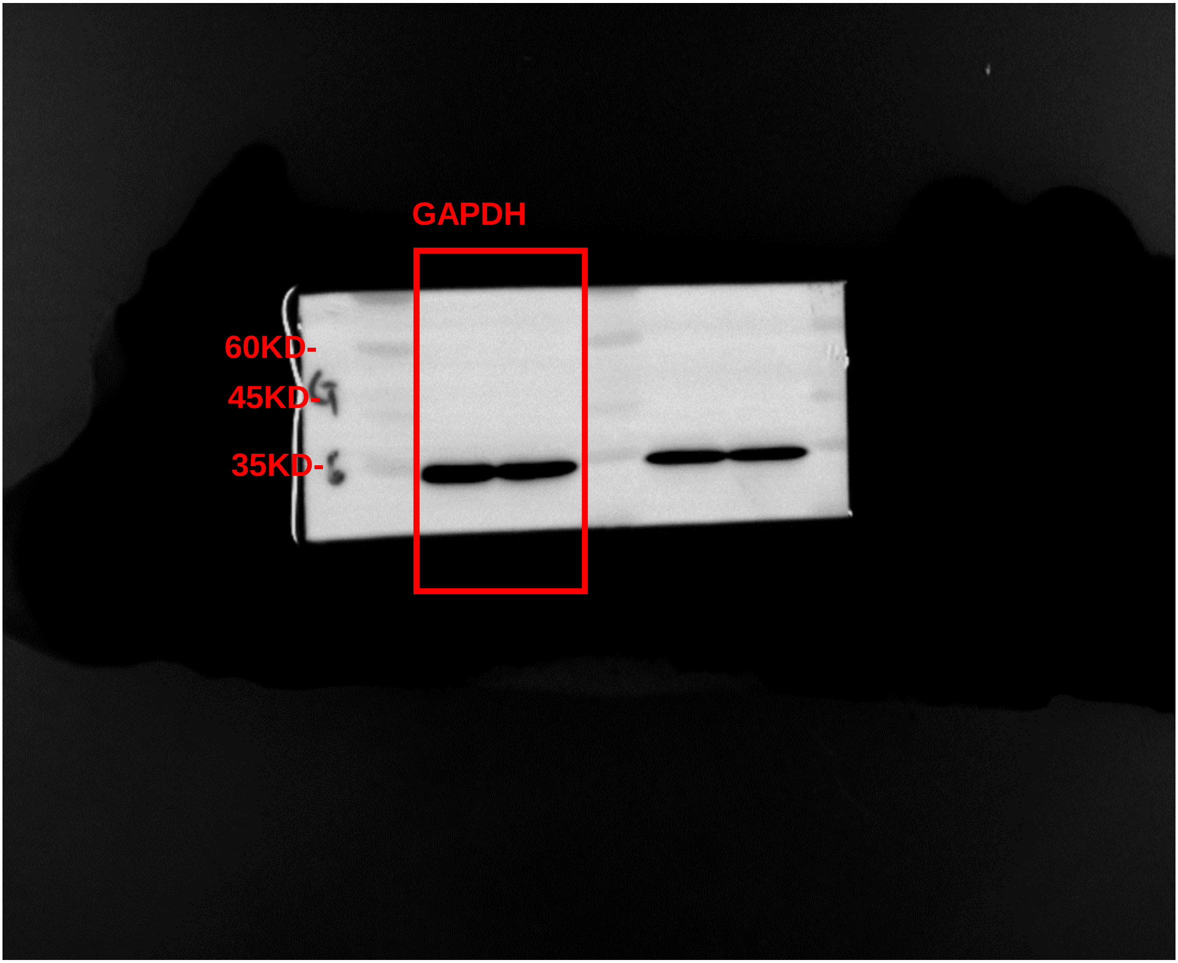

Figure 9A

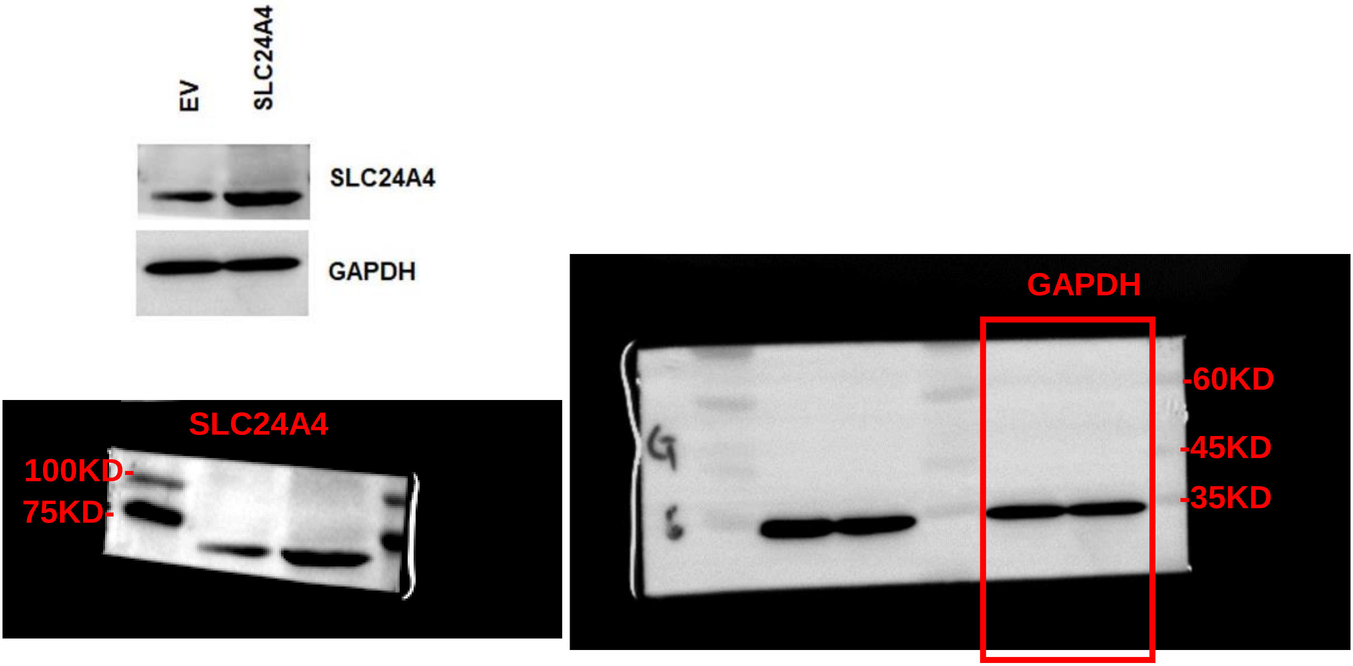
EV
SLC24A4
SLC24A4
GAPDH
SLC24A4
100KD-
75KD-
GAPDH
-60KD
-45KD
-35KD

Figure 9A

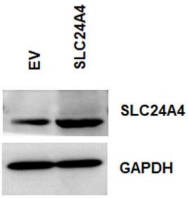

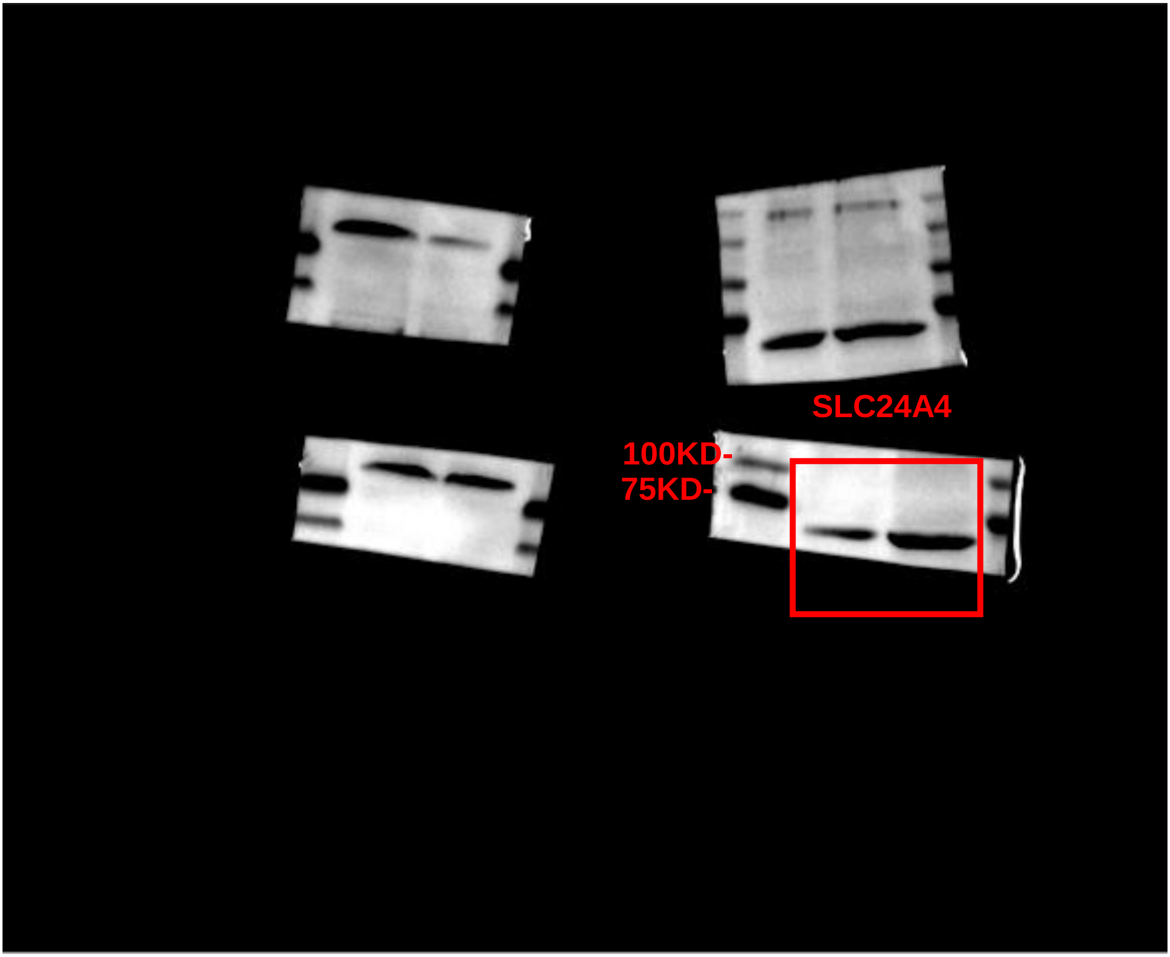

Figure 9A

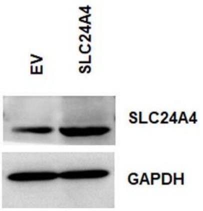

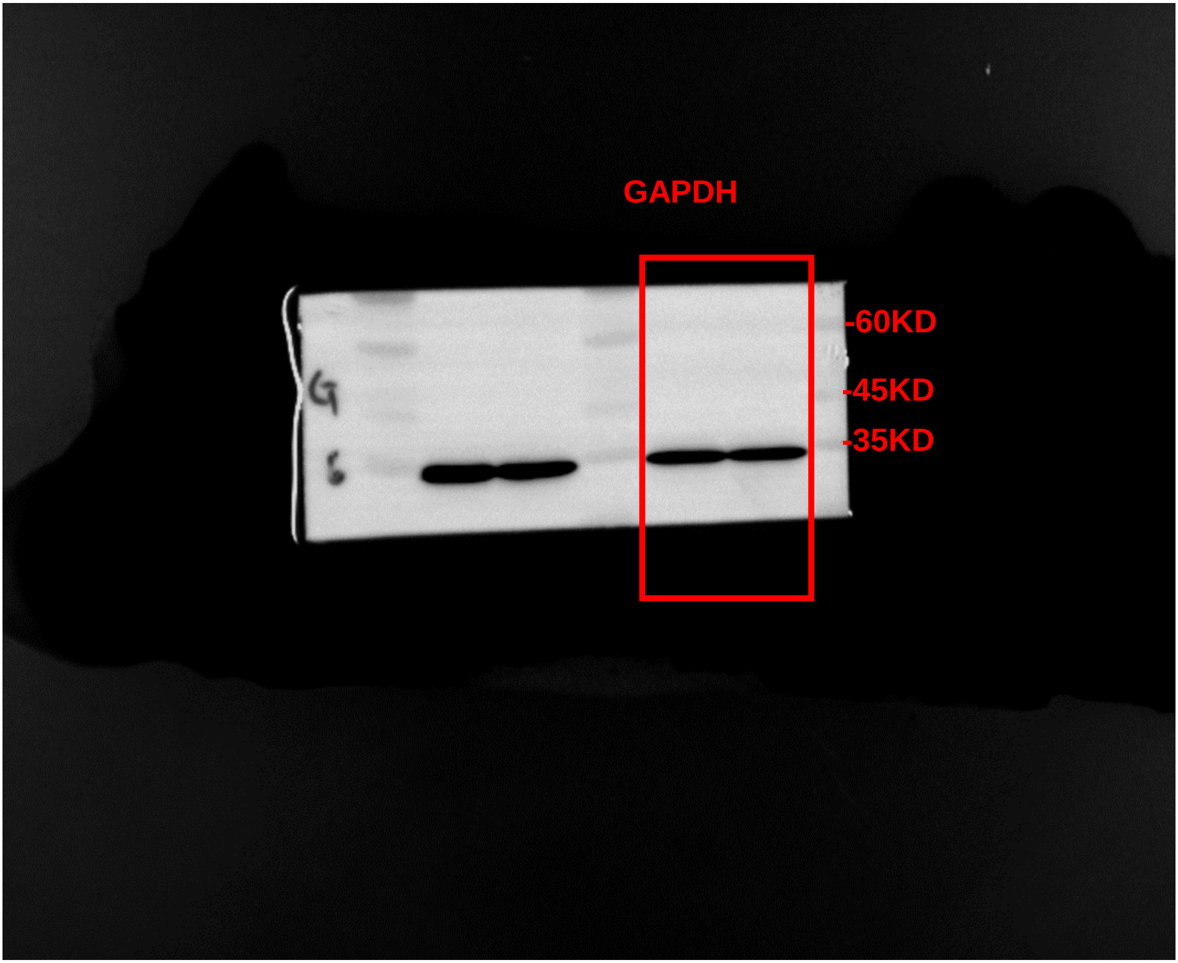

Figure S11A

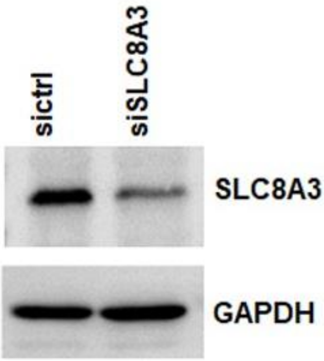

sictrl
siSLC8A3
SLC8A3
GAPDH

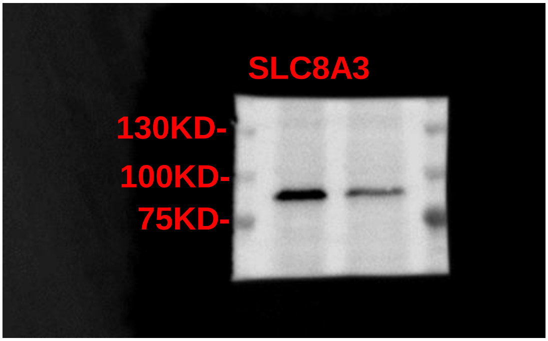

SLC8A3
130KD-
100KD-
75KD-

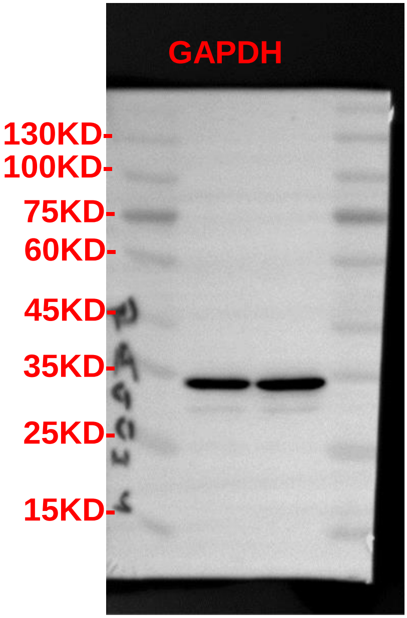

GAPDH
130KD-
100KD-
75KD-
60KD-
45KD-
35KD-
25KD-
15KD-

Figure S11A

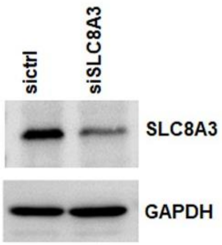

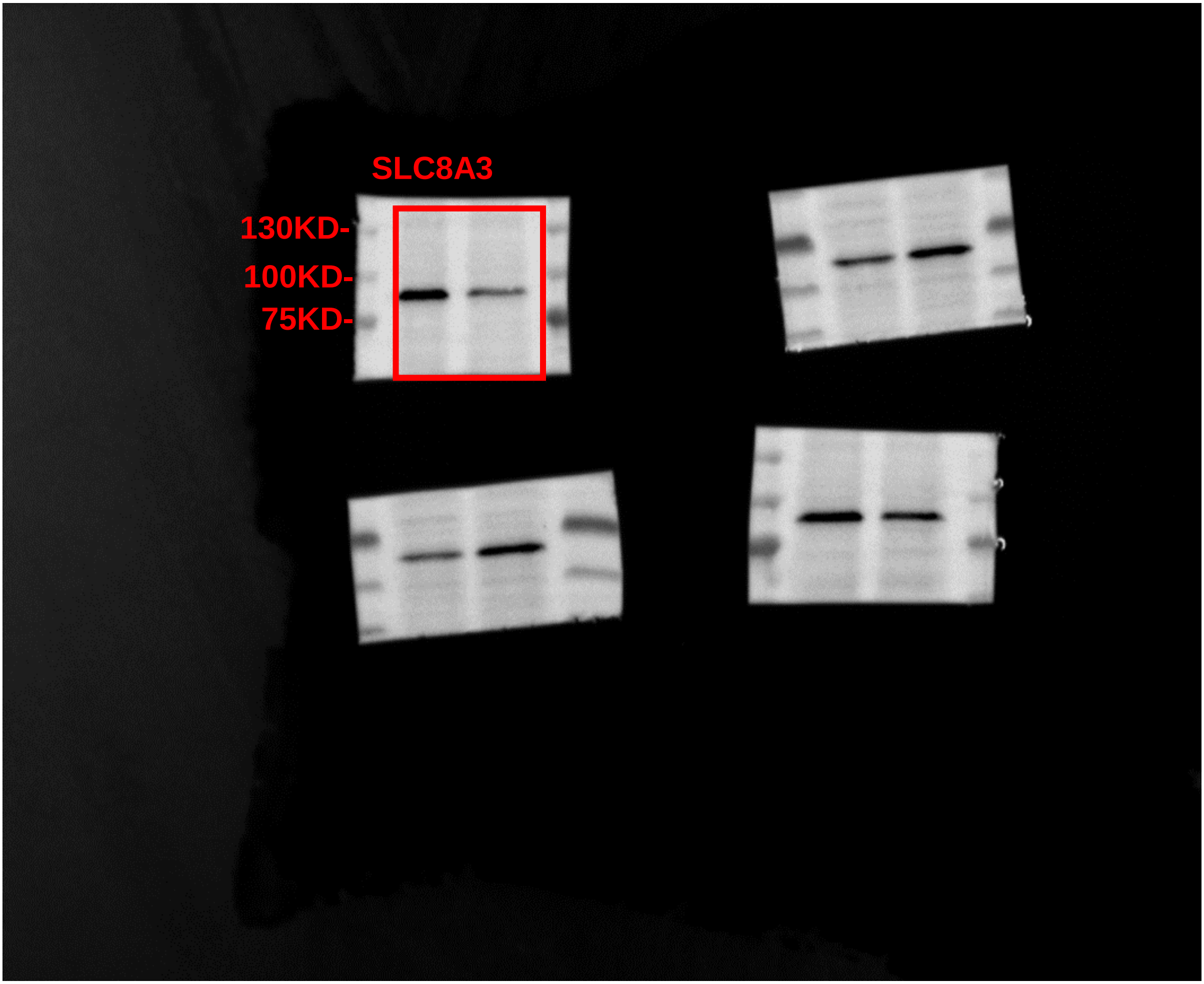

Figure S11A

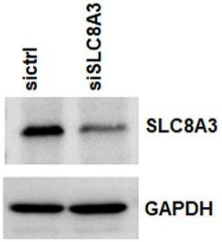

sictrl
siSLC8A3
SLC8A3
GAPDH

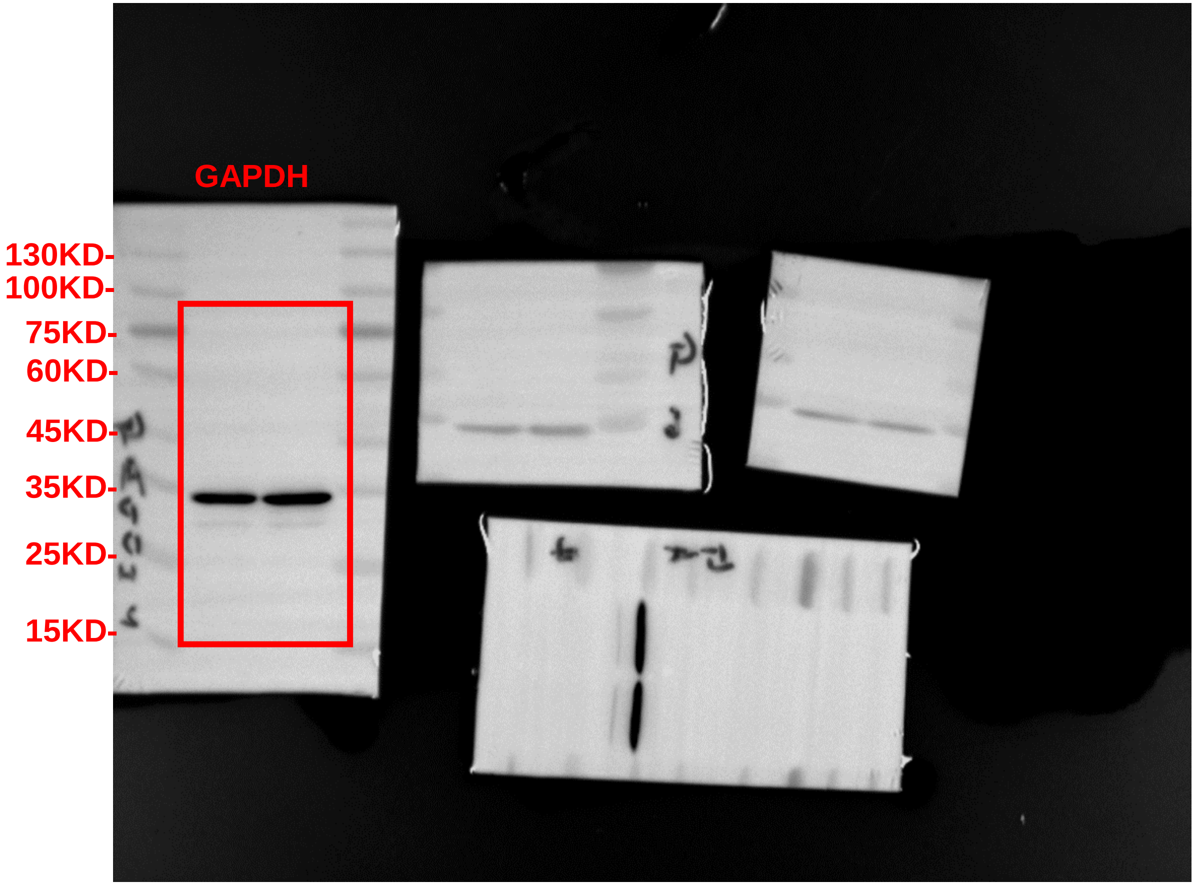

GAPDH
130KD-
100KD-
75KD-
60KD-
45KD-
35KD-
25KD-
15KD-

Figure S11A

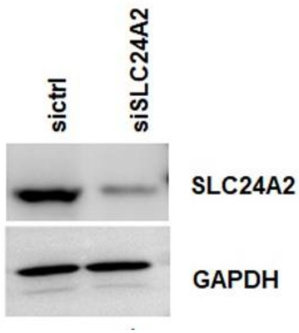
sictrl
siSLC24A2
SLC24A2
GAPDH

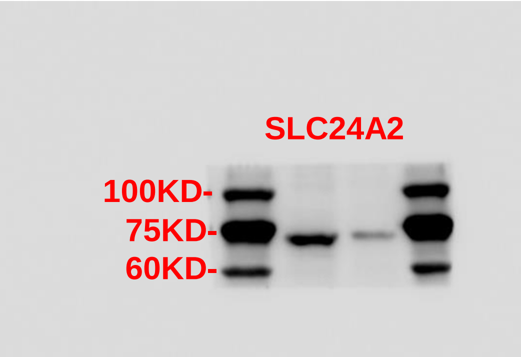
SLC24A2
100KD-
75KD-
60KD-

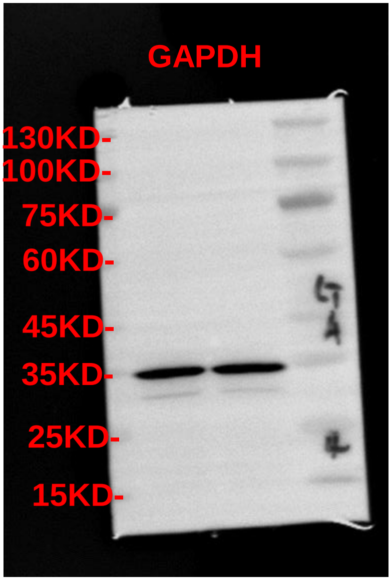
GAPDH
130KD-
100KD-
75KD-
60KD-
45KD-
35KD-
25KD-
15KD-

Figure S11A

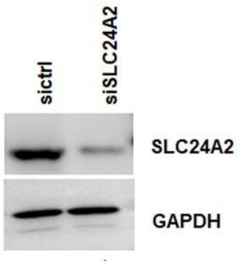

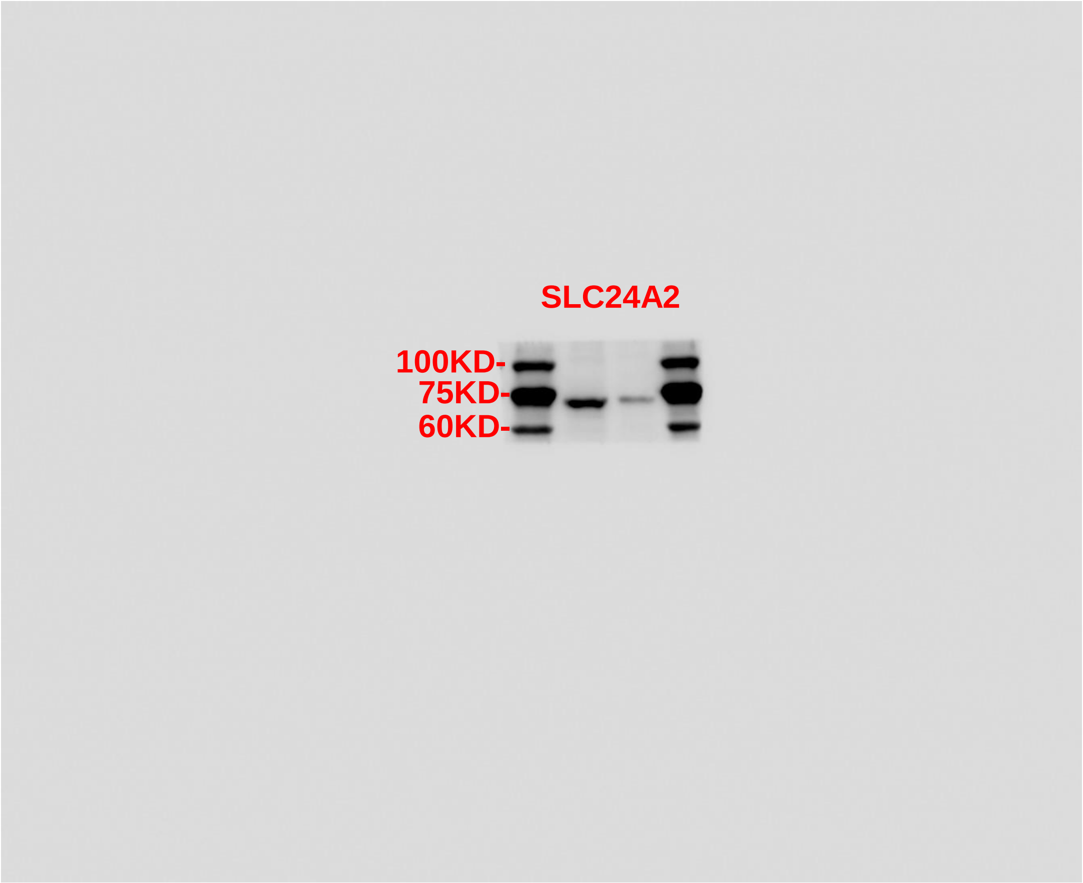

Figure S11A

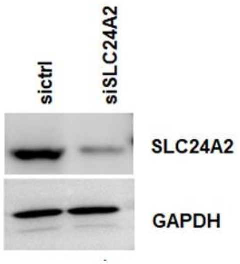
sictrl
siSLC24A2
SLC24A2
GAPDH

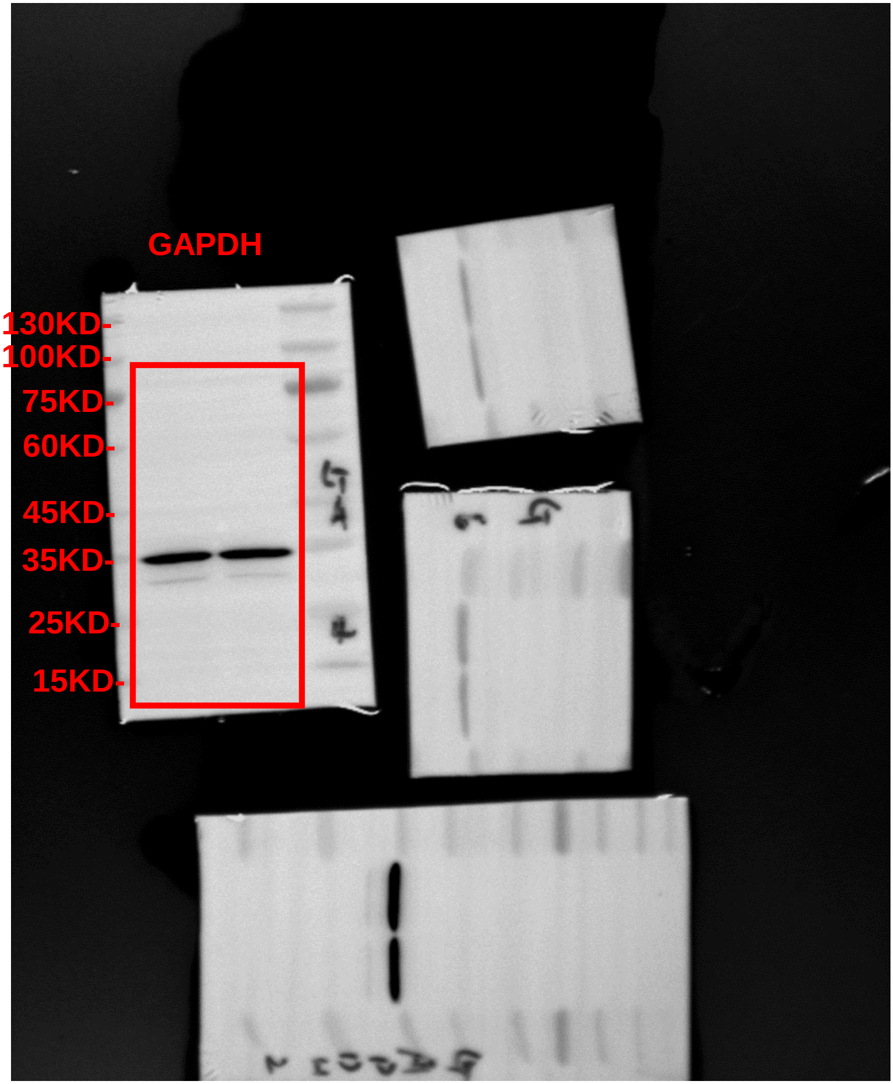
GAPDH
130KD-
100KD-
75KD-
60KD-
45KD-
35KD-
25KD-
15KD-

Figure S11A

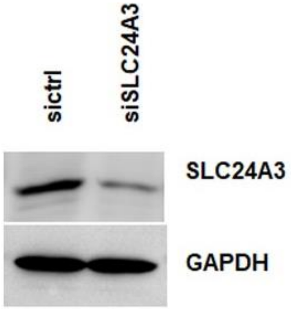
sictrl
siSLC24A3
SLC24A3
GAPDH

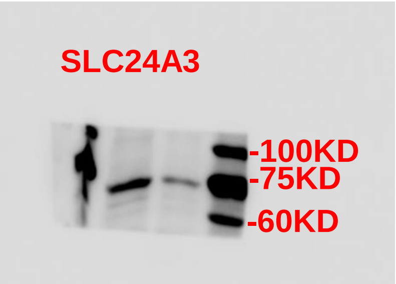
SLC24A3
-100KD
-75KD
-60KD

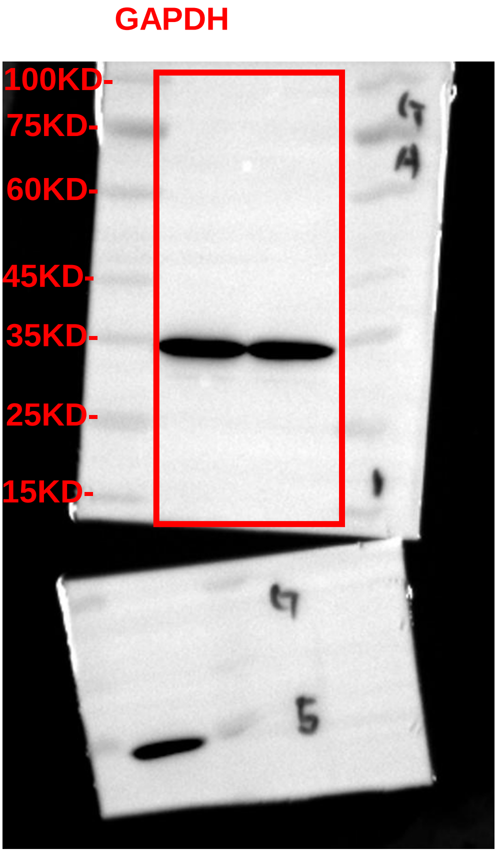
GAPDH
100KD-
75KD-
60KD-
45KD-
35KD-
25KD-
15KD-

Figure S11A

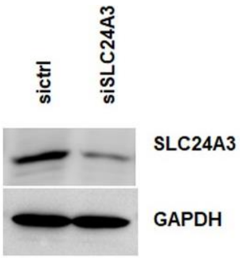

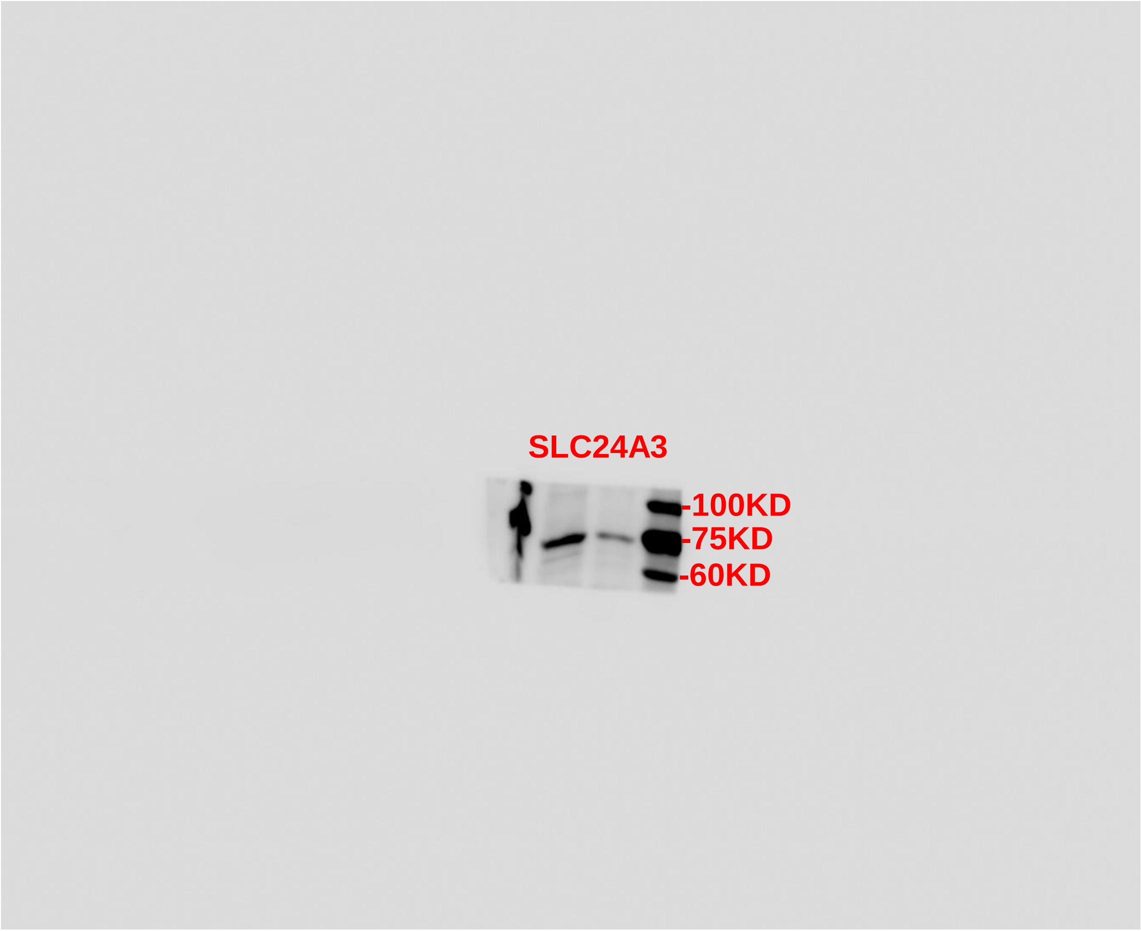

Figure S11A

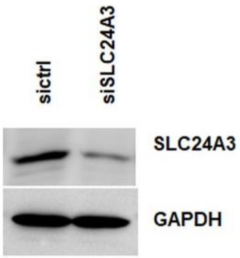
sictrl
siSLC24A3
SLC24A3
GAPDH

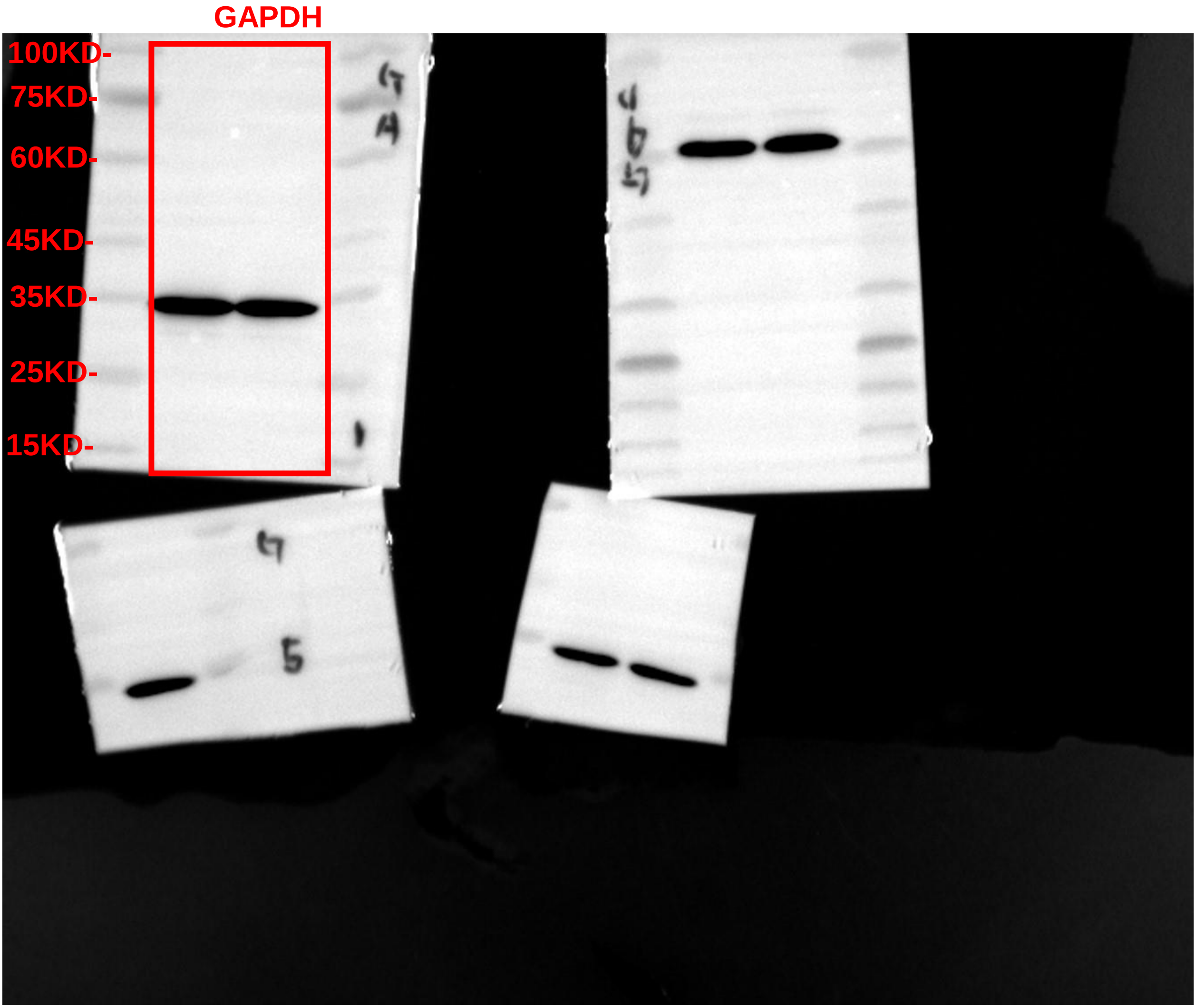
GAPDH
100KD-
75KD-
60KD-
45KD-
35KD-
25KD-
15KD-

Figure S11A

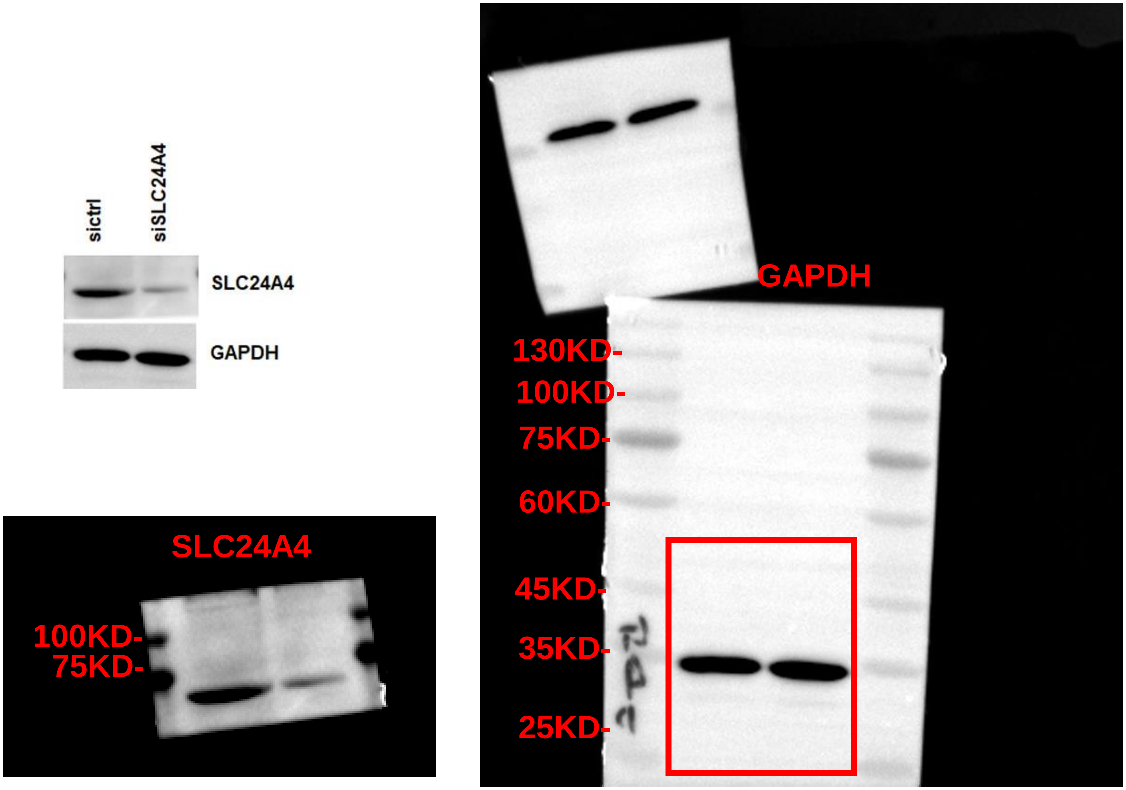
sictrl
siSLC24A4
SLC24A4
GAPDH
SLC24A4
100KD-
75KD-
GAPDH
130KD-
100KD-
75KD-
60KD-
45KD-
35KD-
25KD-

Figure S11A

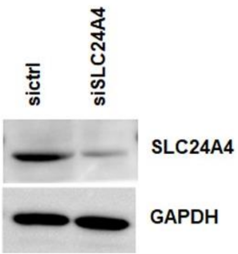

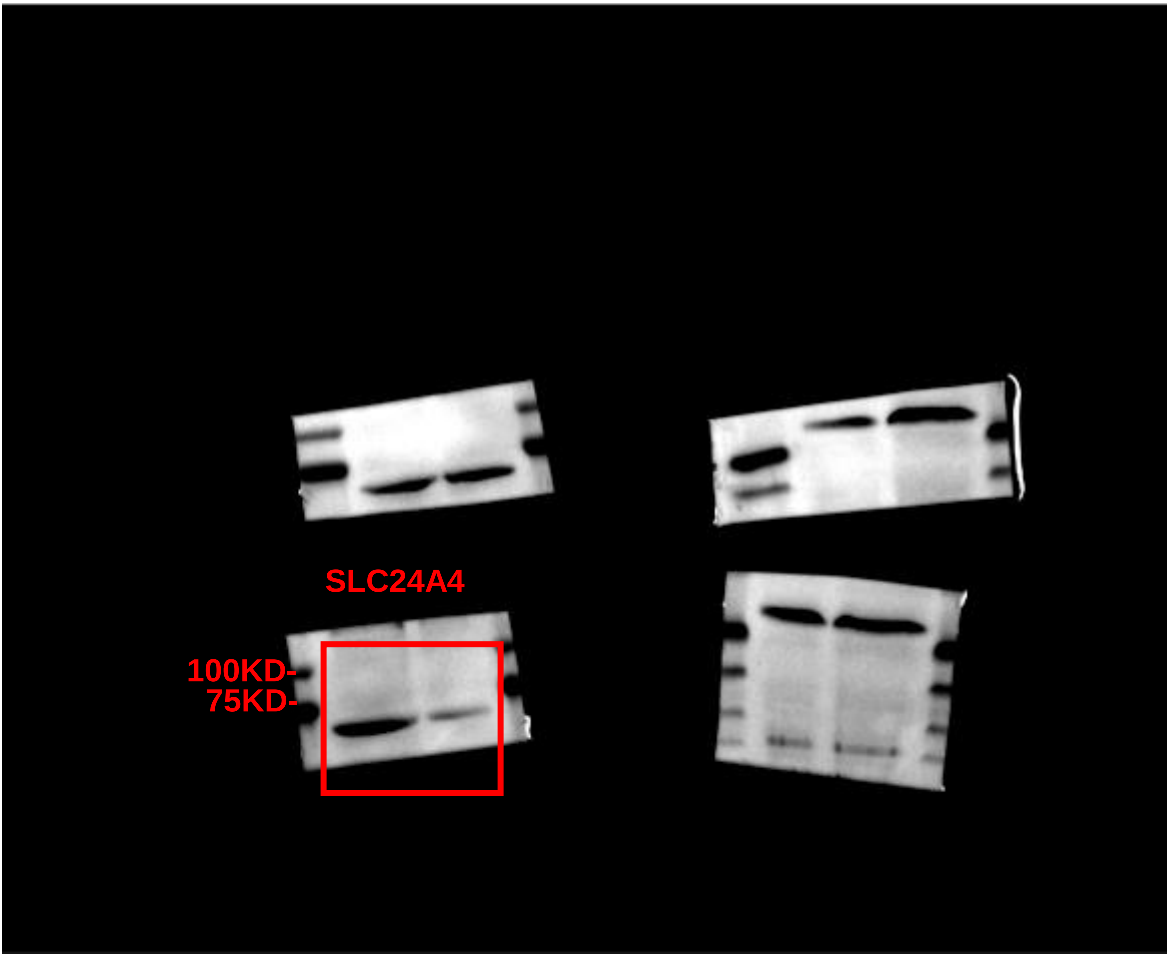

Figure S11A

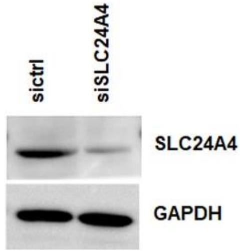

sictrl
siSLC24A4
SLC24A4
GAPDH

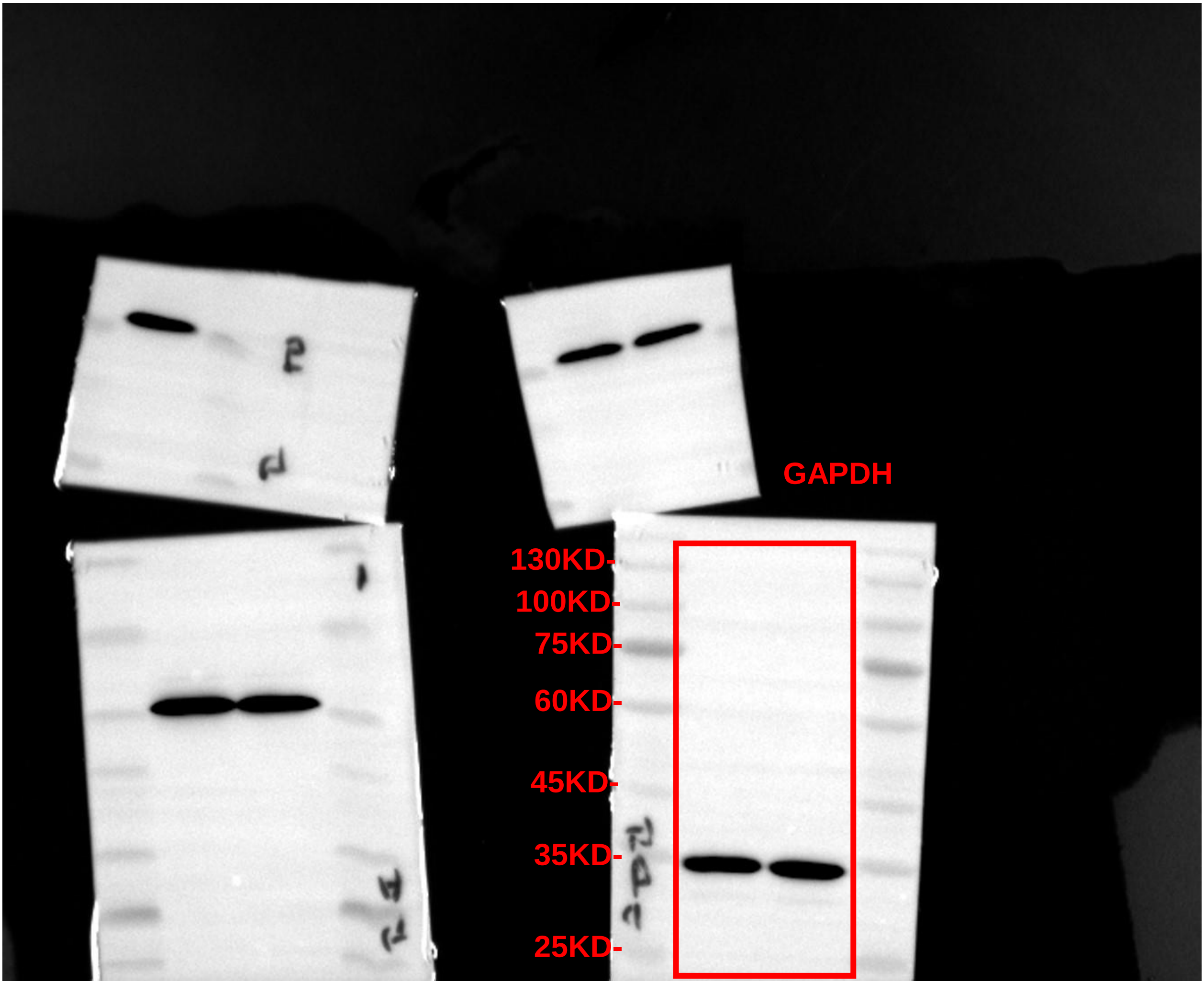

GAPDH
130KD-
100KD-
75KD-
60KD-
45KD-
35KD-
25KD-
