# Supplementary material for: Identification and validation of calcium extrusion-related genes prognostic signature in colon adenocarcinoma
Source: PeerJ. 2024 Jul 10;12:e17582. doi: 10.7717/peerj.17582 (PMC11246022; doi:10.7717/peerj.17582)
Supplement: Figure S5 [file peerj-12-17582-s005.pdf]

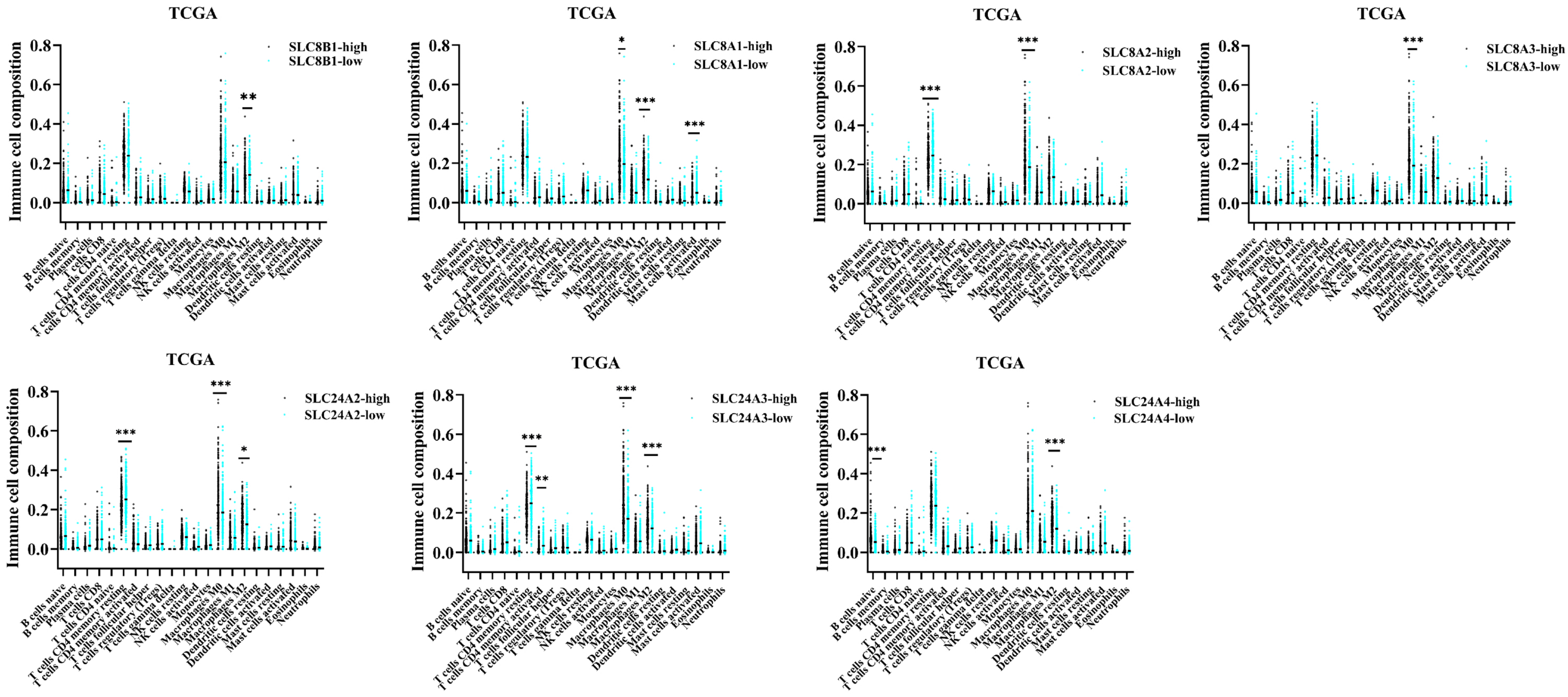

TCGA
Immune cell composition
0.0
0.2
0.4
0.6
0.8
SLC8B1-high
SLC8B1-low
SLC8A1-high
SLC8A1-low
SLC8A2-high
SLC8A2-low
SLC8A3-high
SLC8A3-low
SLC24A2-high
SLC24A2-low
SLC24A3-high
SLC24A3-low
SLC24A4-high
SLC24A4-low
B cells naive
B cells memory
Plasma cells
T cells CD8
T cells CD4 naive
T cells CD4 memory resting
T cells CD4 memory activated
T cells follicular helper
T cells regulatory (Tregs)
T cells gamma delta
NK cells resting
NK cells activated
Monocytes
Macrophages M0
Macrophages M1
Macrophages M2
Dendritic cells resting
Dendritic cells activated
Mast cells resting
Mast cells activated
Eosinophils
Neutrophils
